# Development of nationally representative exposure factor database for children’s products in Korea

**DOI:** 10.1038/s41370-024-00654-1

**Published:** 2024-02-29

**Authors:** Jiyun Shin, Kiyoung Lee, Seung Yeol Park, Miyoung Lim

**Affiliations:** 1 Department of Environmental Health Sciences, Graduate School of Public Health, Seoul National University, Seoul, Republic of Korea; 2 Institute of Health and Environment, Seoul National University, Seoul, Republic of Korea; 3 KSTAT Research, Seoul, Republic of Korea

**Keywords:** Children’s product, Exposure factor, Infant, Children’s exposure, Risk assessment

## Abstract

**Background:**

Children may be exposed to harmful chemicals from their products. Accurate exposure factors are critical for exposure assessment of children’s products. Product usage pattern parameters are relatively limited compared with the chemical concentration, children’s physiological and behavioral parameters.

**Objective:**

The aim of this study was to determine nationally representative Korean exposure factors for the usage patterns of children’s products by sex, age, and season.

**Methods:**

Using proportional quota sampling, a survey of 10,000 households with children aged 0–12 years was conducted twice, once in summer and winter. The children’s ages were divided into four groups: infant (0–2 years old), toddler (3–6), lower-grade elementary student (7–9), and higher-grade elementary student (10–12). Data on exposure factors such as use rate, use frequency, and use duration of 57 children’s products were collected.

**Results:**

The 57 products were classified into five categories: baby products (13), toys (12), daily products (10), sporting goods (8), and stationery (14). The use rates of products in the daily products and stationery category were >90% in both seasons. Two of the 57 products showed significant sex differences in all three exposure factors (*p* < 0.001). Twenty-five of the 44 non-baby products showed significant age differences for all three exposure factors. Twenty-three of the 57 products varied significantly with season for all three exposure factors.

**Impact:**

- This study generated a nationally representative exposure factor database for the usage patterns of children’s products in Korea. The exposure factors for 57 children’s products were investigated through twice survey with quota sampling with each 10,000 children nationwide. Sex, age, and seasonal differences for children’s products were identified. These accurate exposure factors by sex, age, and season can be used as input parameters for refined exposure assessment.

## Introduction

Children’s products and toys may contain chemicals that are potentially harmful to the health and development of infants, toddlers, and children under the age of 13 [1–3]. The chemicals included in children’s products are mainly used as plasticizers, flame retardants, stabilizers, colorants, and fragrances [4, 5]. Because children have different behavioral characteristics from adults, such as hand-to-mouth behavior and playing on the ground [6], they are more easily exposed to harmful chemicals. As immature individuals whose growth and development have not been fully achieved [7, 8], their immature organ systems, rapid organ and tissue growth, higher metabolic rates, and greater surface area to body weight ratios make children more sensitive to exposure to chemicals than adults [9]. Therefore, managing their exposure to harmful substances in children’s products is necessary.

The Korean government implemented the Environmental Health Act with the intent to protect the health of children as sensitive classes based on risk assessment results [10]. Article 24 of the Environmental Health Act stipulates the execution of risk assessments for children’s products to manage hazardous substances affecting children’s health and to restrict or prohibit their use when the results exceed the risk criteria. Exposure assessment is an important process for regulating children’s products based on risk assessment.

Accurate input parameters are key elements of exposure estimation. The children’s product exposure algorithm for exposure estimation consists of the (1) concentration characteristics of the chemicals in the product, (2) physiological and behavioral characteristics of the child, and (3) usage pattern characteristics of the products. The concentrations, content, and migration levels of certain harmful substances in children’s products, such as alternative plasticizers [11], polybrominated flame retardants [12, 13], metal elements [14, 15], and chlorinated paraffins [16], have steadily been studied. Additionally, research has been conducted on the physiological and behavioral parameters of children; their object-to-mouth frequency was monitored by video observation [17–20]. However, resources on product usage pattern parameters are relatively limited compared with the other parameters.

In the United States (US), the “Child-Specific Exposure Factors Handbook” by the Environmental Protection Agency [21] provides recommendations on child-specific exposure factor information, including body weight, non-dietary ingestion factors, and consumer products. Exposure factors for product usage patterns, such as use frequency and amount, exist only for cosmetic and personal care baby products, which are liquid chemical products.

In the European Union (EU), the “Children’s Toys Fact Sheet” by the Dutch National Institute for Public Health and the Environment (RIVM) [22] includes information on mouthing, hand-mouth contact, skin contact frequency, and contact duration of specific solid products, such as teethers, modeling clay, ballpoint pens, and tent canvases [23]. The parameter values of the toys fact sheet are chosen based on the principle of reasonable worst-case estimation and data reliability. The RIVM suggested a quality factor (Q-factor) for all exposure factor parameter values [24, 25]; low Q-factors indicate insufficient or single data sources and high Q-factors indicate sufficient or good quality and relevant data sources. Most of the product usage pattern parameter values in the toys fact sheet had a low Q-factor, and the single values were derived from the estimation of the worst-case assumption.

In Korea, children’s physiological and behavioral exposure factors have been developed using direct measurements and questionnaire surveys [26, 27]. Daily use duration, time and frequency of object-to-hand contact, and time and frequency of object-to-mouth contact were reported for five specific products: toy block, plastic toy, coated wooden toy, play mat, and oil pastel [28]. The product usage pattern information of many other products that children often use in daily life have yet to be assessed.

In the exposure estimation for product risk assessment, input exposure factor values represent the population group. Children’s age groups and sex were considered for the development of a representative exposure factor database on product usage patterns. The use rate, frequency, and time of products may differ according to the demographic characteristics of children. Children’s preferences for several toys differed according to their age [29] and sex [30, 31] in previous studies. Moreover, girls showed higher object-to-mouth frequencies than boys in observation study [32]. Sex differences in mouthing behavior had been reported to be related to differences in outdoor activities [21, 28] in previous studies. Outdoor activities and seasons are known to be associated. Because summer and winter have different temperature and humidity conditions, children’s indoors and outdoors activity patterns vary depending on the season. These changes in activity patterns can also affect the usage patterns of specific children’s products.

This study aimed to establish a national representative exposure factor database for the usage patterns of children’s products in Korea. Considering sex, age, and season, the exposure factors of children’s products were investigated through twice survey with quota sampling with each 10,000 Korean children nationwide.

## Materials and methods

### Sample population

To determine the Korean national representative exposure factors for children’s products, a proportional sampling strategy was designed based on the resident registration statistics by sex, age, and administrative region. The number of participants was determined using proportionate quota sampling according to the resident registration statistics from January 2016. The ratio of boys to girls in each age group and region was considered. The age groups were divided into preschoolers (0, 1–2, 3–4, and 5–6 year olds) and elementary school students (7–9 and 10–12 year olds). Eight metropolitan cities (Seoul, Busan, Daegu, Incheon, Gwangju, Daejeon, Ulsan, and Sejong) and nine provinces (Gyeonggi, Gangwon, Chungcheongbuk-do, Chungcheongnam-do, Jeollabuk-do, Jeollanam-do, Gyeongsangbuk-do, Gyeongsangnam-do, and Jeju) of the Korean regional divisions were included, with some provinces being subdivided into cities (Korean administrative division unit, si) and counties (Korean administrative division unit, gun).

A total of 40,000 panels with children aged 0–12 were recruited by KSTAT Research (Seoul, Republic of Korea) divided into 300 subgroups (2 sexes, 6 ages, and 25 regions). The survey was conducted twice on 10,000 households in the recruitment panel, once in the summer (July–October 2017) and winter (February–March 2018) by controlled quota sampling. The demographic proportions of the quotas for the 10,000 respondents in a single survey are presented in Table 1. Among the recruited panel, participants who reaffirmed their intentions were selected based on the set quotas. The survey of the subgroups was carried out until the number of respondents fill the quota.

**Table 1:** Number of respondents for single surveys in summer and winter by children’s sex, age, and regional quota.

		0 yrs		1–2 yrs		3–4 yrs		5–6 yrs		7–9 yrs		10–12 yrs		Total
		Boy	Girl	Boy	Girl	Boy	Girl	Boy	Girl	Boy	Girl	Boy	Girl	
Seoul		56	54	79	77	81	79	79	77	98	96	99	95	970
Busan		32	31	46	45	48	47	47	45	57	55	58	55	566
Daegu		27	27	40	39	42	41	41	40	52	50	53	51	503
Incheon		32	31	46	45	48	47	47	46	58	56	57	56	569
Gwangju		22	21	33	32	35	34	35	34	44	42	44	42	418
Daejeon		23	22	33	33	35	34	34	33	43	42	43	42	417
Ulsan		21	21	31	30	32	31	31	30	38	36	37	35	373
Sejong		11	10	15	15	16	16	16	16	19	19	18	18	189
Gyeonggi	City	66	65	97	95	103	100	101	98	125	122	123	120	1215
	County	7	7	11	11	11	11	12	11	14	14	15	14	138
Gangwon	City	18	17	26	26	28	28	28	28	35	34	36	35	339
	County	10	10	15	14	15	15	16	15	20	19	20	19	188
Chungcheongbuk-do	City	20	20	29	29	31	30	30	30	37	36	38	36	366
	County	10	10	15	15	16	15	16	15	20	19	20	19	190
Chungcheongnam-do	City	25	24	36	35	38	37	37	36	46	44	44	43	445
	County	10	10	15	14	16	15	16	15	20	19	20	20	190
Jeollabuk-do	City	21	21	32	31	34	33	33	33	42	41	43	41	405
	County	9	9	14	14	15	14	15	14	18	17	18	17	174
Jeollanam-do	City	19	18	27	27	29	29	29	28	36	34	36	34	346
	County	15	15	22	21	22	22	22	21	27	26	27	26	266
Gyeongsangbuk-do	City	27	26	39	37	40	39	39	38	48	46	48	46	473
	County	12	12	17	17	18	17	17	16	21	20	21	20	208
Gyeongsangnam-do	City	32	32	47	46	50	48	49	47	60	58	59	56	584
	County	10	10	15	15	16	16	16	16	20	19	20	19	192
Jeju		15	14	22	21	23	23	23	22	29	28	29	27	276
	Total	550	537	802	784	842	821	829	804	1027	992	1026	986	10,000

### Development of questionnaire

The questionnaire inquired about the demographic characteristics of the respondents and their product usage patterns. Children’s products were classified into five categories, namely baby product, toy, daily product, sporting goods, and stationery, according to Established Rule No. 585 in the Guidance on Procedures and Methods for Risk Assessment of Environmental Hazardous Substances of the Korean Ministry of Environment [33]. The 57 children’s products were selected from 5 categories including 13 baby products (self-righting toy, baby rattle, squeaky toy, tactile toy, baby mobile toy, teether, pacifier, baby bouncer, baby walker, diaper, teeth wipes, baby bottle, and baby playpen), 12 toys (play sand, bubble-making toy, kid’s car, kid’s bike, card game, board game, electronic game, toy audio player, toy video player, beach ball, swimming goggles, and bath toy), 10 daily products (wet wipes, toothbrush, cotton swab, towel, handkerchief, food tray, lunch box, water bottle, car seat, and kid’s chair), 8 sporting goods (ball, gloves, bicycle, inline skates, roller shoes, skateboard, kick scooter, and picnic mat), and 14 stationeries (oil pastel, colored pencil, paint supplies, workbook, sticker/sticker book, notebook, ballpoint pen, pencil, marker pen, eraser, correction tape/fluid, glue, adhesive, and scissors).

The questionnaire included questions on the use rate of specific children’s products in the last three months as well as their use frequency (event per day) and use duration (min/event). The target population for children’s products were classified into three children’s age groups: infants aged 0–2, toddlers aged 3–6, and elementary school students aged 7–12.

### Data collection

Trained field survey staff visited each participant and collected their children’s product use information through face-to-face interviews with their parents using the structured questionnaire. The field staff used a script and picture prompts to help the participants with responding to the questions. For elementary school students aged 7–12 years old, children and their parents responded together to the questionnaire. The face-to-face survey was approved by the Institutional Review Board of the Seoul National University (SNU IRB 17-04-072). A total of 20,000 children’s product usage pattern data was collected during the two seasons.

### Data analysis

The mean and standard deviation of the use rate, use frequency, and use duration of all children’s products were calculated. The chi-square test was used to analyze the product use rates by sex, age, and season. An analysis of variance (ANOVA) and *t*-test were used to analyze the use frequency and time by sex, age, and season. All statistical analyses were performed using SPSS ver. 25 (IBM Corp., Armonk, NY, USA).

## Results

### Study population

The participants were 50.8% boys and 49.2% girls. The percentages of infants (0–2 years old), toddlers (3–6 years old), lower-grade elementary student (7–9 years old), and higher-grade elementary student (10–12 years old) were 26.7%, 33.0%, 20.2%, and 20.1%, respectively. The largest number of respondents were from the city area of Gyeonggi (12.2%), Seoul (9.7%), and the city area of Gyeongsangnam-do (5.8%). The places with the least number of respondents were the county areas of Gyeonggi (1.4%), Jeollabuk-do (1.7%), and Gangwon (1.9%). The number of respondents for quotas coincided with the two surveys in summer and winter.

### Use rate

The use rates of each children’s product by season are shown in Table 2 while the use rates of children’s products by sex and age group are shown in Table S1 and Table S2, respectively. In both seasons, the use rates of towels, toothbrushes, and wet wipes in the daily product category, and pencils, notebooks, and erasers in the stationery category were >90%. A significant seasonal difference in the use rate was observed in most of the baby product category. Except for toy video players, the use rate of all products in the toy category significantly differed by age group and the use rate of all products except kid’s bikes varied significantly with the season. In the daily product category, the use rate of all the products significantly differed by age group. Significant sex and age differences in the use rate were observed for all products in the sporting goods category. Finally, in the stationery category, the use rate of all products varied significantly by age group, and except for workbooks, the use rate of all products seasonally differed.

**Table 2:** Target population of children’s products classified into three age groups: infants (0–2 years old, *n* = 2673 for a single survey), toddlers (3–6 years old, *n* = 3296 for a single survey), and elementary school kids (7–12 years, *n* = 4031 for a single survey), and use rates (%) in the summer survey, winter survey, and overall.

Category	Product	Target population			Use rates (%) of target population		
		Infant (0–2 yrs)	Toddler (3–6 yrs)	School kid (7–12 yrs)	Summer	Winter	Overall
Baby product	Self-righting toy	O^a^	-^a^	-^a^	48.4	49.0	48.7
	Baby rattle*	O	-	-	44.1	39.4	41.8
	Squeaky toy	O	-	-	74.1	74.6	74.4
	Tactile toy	O	-	-	74.4	74.2	74.3
	Baby mobile toy*	O	-	-	23.4	15.2	19.3
	Teether*	O	-	-	43.5	36.9	40.2
	Pacifier*	O	-	-	53.0	42.6	47.8
	Baby bouncer*	O	-	-	45.0	25.6	35.3
	Baby walker*	O	-	-	33.5	21.8	27.6
	Diaper*	O	-	-	86.5	82.8	84.6
	Teeth wipes*	O	-	-	34.0	29.7	31.9
	Baby bottle*	O	-	-	75.8	51.7	63.7
	Baby playpen*	O	-	-	42.3	23.5	32.9
Toy	Play sand*	O	O	O	30.8	25.6	28.2
	Bubble-making toy*	O	O	O	60.4	40.0	50.2
	Kid’s car*	O	O	-	50.1	40.7	45.4
	Kid’s bike*	O	O	-	53.3	41.9	47.6
	Card game*	-	O	O	61.5	51.2	56.4
	Board game*	-	O	O	54.5	50.7	52.6
	Electronic game*	-	O	O	27.7	30.9	29.3
	Toy audio player*	O	O	O	47.5	37.8	42.7
	Toy video player	O	O	O	37.3	36.3	36.8
	Beach ball*	O	O	O	67.8	21.8	44.8
	Swimming goggles*	-	O	O	63.0	17.5	40.2
	Bath toy*	O	O	-	74.4	55.2	64.8
Daily product	Wet wipes*	O	O	O	97.1	95.8	96.5
	Toothbrush*	O	O	O	97.1	96.5	96.8
	Cotton swab*	O	O	O	76.6	86.3	81.5
	Towel*	O	O	O	99.7	99.1	99.4
	Handkerchief	O	O	O	47.4	46.1	46.8
	Food tray*	O	O	O	84.2	82.2	83.2
	Lunch box*	O	O	O	57.0	32.9	45.0
	Water bottle*	O	O	O	92.9	83.3	88.1
	Car seat*	O	O	-	80.3	70.3	75.3
	Kid’s chair*	O	O	-	52.0	41.1	46.5
Sporting goods	Ball*	O	O	O	72.2	58.6	65.4
	Gloves*	-	O	O	18.6	15.9	17.3
	Bicycle*	-	O	O	61.5	53.4	57.5
	Inline skates	-	O	O	26.6	26.1	26.3
	Roller shoes*	-	O	O	16.1	10.8	13.5
	Skateboard	-	O	O	7.8	8.1	7.9
	Kick scooter*	-	O	O	58.6	41.9	50.3
	Picnic mat*	O	O	O	65.8	20.0	42.9
Stationery	Oil pastel*	O	O	O	76.9	66.2	71.5
	Colored pencil*	O	O	O	85.1	75.6	80.4
	Paint supplies*	O	O	O	29.6	15.6	22.6
	Workbook	O	O	O	71.2	71.1	71.1
	Sticker/sticker book*	O	O	O	63.4	55.4	59.4
	Notebook*	-	O	O	93.6	90.7	92.2
	Ballpoint pen*	-	O	O	41.0	44.3	42.7
	Pencil*	-	O	O	95.8	93.3	94.5
	Marker pen*	-	O	O	58.6	45.7	52.2
	Eraser*	-	O	O	94.2	90.3	92.2
	Correction tape/fluid*	-	O	O	14.3	20.3	17.3
	Glue*	-	O	O	91.8	77.7	84.8
	Adhesive*	-	O	O	13.1	11.0	12.1
	Scissors*	-	O	O	96.0	85.7	90.8

*Products with significant seasonal differences in the chi-square test (*p* ≤ 0.001).

^a^Products’ survey target population were marked as “O”, and non-target population were marked as “-”.

### Use frequency

The use frequency of each children’s product by season is shown in Table 3. The use frequencies of children’s products by sex and age group are shown in Table S3 and Table S4, respectively and their percentiles are shown in Table S5 and Table S6, respectively. The product with the highest use frequency in one day was the diaper. Products used more than once a day were mainly from the daily product and baby product categories. All other products were used more than once a week, except for swimming goggles, play sand, bubble-making toys, lunch boxes, and picnic mats. The use frequency of most products in the baby products, toys, daily products, and stationery categories did not significantly vary by sex. Meanwhile, the use frequency of most non-baby products (85.0%) was significantly different by age group. Seasonal differences were also significant for most of the products (83.0%).

**Table 3:** Use frequency of children’s product in the summer survey, winter survey, and overall.

Category	Product	Summer (AM ± STD, event/day)	Winter (AM ± STD, event/day)	Overall (AM ± STD, event/day)
Baby product	Self-righting toy*	1.55 ± 1.42	1.31 ± 1.39	1.43 ± 1.41
	Baby rattle	1.97 ± 1.96	1.93 ± 1.83	1.95 ± 1.90
	Squeaky toy*	1.63 ± 1.51	1.29 ± 1.25	1.46 ± 1.39
	Tactile toy*	1.54 ± 1.39	1.31 ± 1.45	1.43 ± 1.42
	Baby mobile toy	1.55 ± 1.56	1.74 ± 1.53	1.63 ± 1.55
	Teether*	2.39 ± 1.91	1.86 ± 1.57	2.15 ± 1.78
	Pacifier*	3.01 ± 2.03	2.54 ± 1.81	2.80 ± 1.95
	Baby bouncer*	2.04 ± 1.48	1.38 ± 1.26	1.80 ± 1.44
	Baby walker	2.09 ± 1.73	2.10 ± 1.58	2.10 ± 1.67
	Diaper*	6.48 ± 2.90	5.60 ± 2.78	6.05 ± 2.87
	Teeth wipes*	1.77 ± 0.99	1.33 ± 0.96	1.57 ± 1.00
	Baby bottle*	3.83 ± 1.94	3.63 ± 1.86	3.75 ± 1.91
	Baby playpen*	1.79 ± 1.47	1.45 ± 1.41	1.66 ± 1.46
Toy	Play sand	0.13 ± 0.19	0.12 ± 0.13	0.12 ± 0.17
	Bubble-making toy*	0.10 ± 0.18	0.08 ± 0.13	0.09 ± 0.16
	Kid’s car*	1.01 ± 1.22	0.85 ± 1.03	0.94 ± 1.14
	Kid’s bike*	0.55 ± 0.56	0.36 ± 0.49	0.47 ± 0.54
	Card game*	0.48 ± 0.71	0.37 ± 0.54	0.43 ± 0.64
	Board game*	0.24 ± 0.37	0.18 ± 0.27	0.22 ± 0.33
	Electronic game*	0.42 ± 0.56	0.37 ± 0.47	0.39 ± 0.52
	Toy audio player*	1.01 ± 1.04	0.91 ± 1.03	0.96 ± 1.04
	Toy video player*	0.89 ± 0.91	0.72 ± 0.88	0.80 ± 0.90
	Beach ball*	0.10 ± 0.26	0.28 ± 0.52	0.15 ± 0.35
	Swimming goggles*	0.11 ± 0.23	0.23 ± 0.44	0.14 ± 0.29
	Bath toy*	0.64 ± 0.50	0.45 ± 0.49	0.56 ± 0.50
Daily product	Wet wipes*	4.46 ± 3.82	3.17 ± 2.88	3.82 ± 3.45
	Toothbrush*	2.48 ± 0.65	2.39 ± 0.65	2.44 ± 0.65
	Cotton swab*	0.41 ± 0.52	0.33 ± 0.42	0.37 ± 0.47
	Towel	2.92 ± 1.38	2.94 ± 1.23	2.93 ± 1.31
	Handkerchief*	2.36 ± 2.68	1.59 ± 2.15	1.98 ± 2.46
	Food tray*	1.30 ± 0.92	1.11 ± 0.89	1.20 ± 0.91
	Lunch box*	0.06 ± 0.24	0.07 ± 0.20	0.07 ± 0.22
	Water bottle*	1.84 ± 2.13	1.36 ± 1.59	1.61 ± 1.91
	Car seat*	0.51 ± 0.61	0.36 ± 0.46	0.44 ± 0.55
	Kid’s chair	1.91 ± 1.27	1.90 ± 1.19	1.90 ± 1.24
Sporting goods	Ball*	0.39 ± 0.72	0.35 ± 0.65	0.37 ± 0.69
	Gloves*	0.25 ± 0.49	0.15 ± 0.16	0.20 ± 0.38
	Bicycle*	0.41 ± 0.50	0.30 ± 0.40	0.36 ± 0.46
	Inline skates*	0.21 ± 0.24	0.17 ± 0.26	0.19 ± 0.25
	Roller shoes*	0.53 ± 0.79	0.45 ± 0.74	0.50 ± 0.77
	Skateboard*	0.20 ± 0.29	0.13 ± 0.17	0.16 ± 0.24
	Kick scooter*	0.39 ± 0.44	0.29 ± 0.42	0.35 ± 0.43
	Picnic mat*	0.06 ± 0.13	0.08 ± 0.23	0.06 ± 0.16
Stationery	Oil pastel*	0.38 ± 0.43	0.31 ± 0.46	0.35 ± 0.44
	Colored pencil*	0.52 ± 0.65	0.40 ± 0.53	0.46 ± 0.60
	Paint supplies*	0.27 ± 0.39	0.20 ± 0.30	0.25 ± 0.36
	Workbook*	0.73 ± 0.78	0.68 ± 0.79	0.70 ± 0.78
	Sticker/sticker book*	0.39 ± 0.50	0.29 ± 0.40	0.35 ± 0.46
	Notebook	NA	NA	NA
	Ballpoint pen	NA	NA	NA
	Pencil	NA	NA	NA
	Marker pen	NA	NA	NA
	Eraser	6.0 ± 9.9	5.3 ± 4.9	5.7 ± 7.9
	Correction tape/fluid	2.8 ± 3.1	2.8 ± 1.6	2.8 ± 2.3
	Glue*	3.4 ± 3.6	3.6 ± 3.0	3.5 ± 3.4
	Adhesive*	2.4 ± 2.7	2.9 ± 3.1	2.6 ± 2.9
	Scissors	3.7 ± 4.4	3.8 ± 3.4	3.7 ± 3.9

*AM* arithmetic mean, *STD* standard deviation.

*Products with significant seasonal differences in the *t* test (*p* ≤ 0.001).

### Use duration

The use duration of each children’s product by season is shown in Table 4. The use durations of children’s products by sex and age group are shown in Table S7 and Table S8, respectively and their percentiles are shown in Table S9 and Table S10, respectively. The product with the highest use duration in one day was the diaper. Products used for >30 min a day were mainly from the sporting goods and toy categories. Products used for <5 min a day were mostly from the daily products and stationery categories. The use durations did not significantly vary by sex for most products in the baby product, toy, and daily product categories. Meanwhile, the use frequency of all non-baby products was significantly different by age group. Seasonal differences were also significant for most of the products (75.0%).

**Table 4:** Use durations of children’s product in the summer survey, winter survey, and overall.

Category	Product	Summer (AM ± STD, min/event)	Winter (AM ± STD, min/event)	Overall (AM ± STD, min/event)
Baby product	Self-righting toy*	9.3 ± 6.3	8.7 ± 5.5	9.0 ± 6.0
	Baby rattle*	8.7 ± 7.0	7.4 ± 5.0	8.1 ± 6.2
	Squeaky toy	11.3 ± 8.3	11.4 ± 7.2	11.3 ± 7.7
	Tactile toy*	10.8 ± 8.1	12.2 ± 8.1	11.5 ± 8.1
	Baby mobile toy*	8.5 ± 5.9	10.4 ± 7.5	9.2 ± 6.7
	Teether*	7.7 ± 6.5	10.4 ± 7.5	9.0 ± 7.1
	Pacifier	17.7 ± 11.9	17.4 ± 11.2	17.6 ± 11.6
	Baby bouncer*	15.6 ± 7.4	14.4 ± 6.8	15.2 ± 7.2
	Baby walker*	19.1 ± 8.9	15.6 ± 8.3	17.7 ± 8.8
	Diaper	184.3 ± 125.2	188.2 ± 91.0	186.2 ± 109.9
	Teeth wipes*	1.9 ± 1.2	2.1 ± 1.7	2.0 ± 1.5
	Baby bottle*	14.0 ± 6.6	13.3 ± 6.2	13.7 ± 6.5
	Baby playpen*	28.8 ± 21.7	25.4 ± 19.1	27.6 ± 20.9
Toy	Play sand	22.7 ± 14.4	22.1 ± 12.1	22.4 ± 13.4
	Bubble-making toy*	20.7 ± 11.6	17.4 ± 10.4	19.4 ± 11.3
	Kid’s car*	18.1 ± 10.2	15.9 ± 9.0	17.1 ± 9.8
	Kid’s bike*	23.4 ± 9.8	21.6 ± 9.8	22.6 ± 9.8
	Card game	23.3 ± 12.5	22.8 ± 11.9	23.1 ± 12.3
	Board game*	32.3 ± 18.3	30.8 ± 16.0	31.6 ± 17.3
	Electronic game*	34.9 ± 21.4	33.0 ± 20.1	33.9 ± 20.7
	Toy audio player*	15.6 ± 8.9	14.8 ± 8.4	15.2 ± 8.7
	Toy video player*	17.8 ± 9.6	16.7 ± 8.7	17.3 ± 9.2
	Beach ball*	28.4 ± 21.3	19.3 ± 14.1	26.2 ± 20.1
	Swimming goggles	41.5 ± 25.4	41.5 ± 20.2	41.5 ± 24.4
	Bath toy*	16.2 ± 9.0	13.6 ± 8.2	15.1 ± 8.8
Daily product	Wet wipes	NA	NA	NA
	Toothbrush*	2.8 ± 1.2	2.7 ± 1.3	2.7 ± 1.3
	Cotton swab*	1.8 ± 1.6	2.0 ± 1.7	1.9 ± 1.6
	Towel*	3.4 ± 3.1	3.4 ± 3.5	3.4 ± 3.3
	Handkerchief*	5.1 ± 16.1	7.6 ± 24.0	6.3 ± 20.4
	Food tray	NA	NA	NA
	Lunch box	NA	NA	NA
	Water bottle	NA	NA	NA
	Car seat*	32.9 ± 17.3	35.8 ± 22.0	34.3 ± 19.7
	Kid’s chair*	19.0 ± 7.8	18.1 ± 8.9	18.6 ± 8.3
Sporting goods	Ball	24.2 ± 15.9	23.8 ± 15.0	24.0 ± 15.5
	Gloves*	36.4 ± 21.6	34.5 ± 16.4	35.5 ± 19.4
	Bicycle*	35.0 ± 17.1	31.7 ± 14.6	33.5 ± 16.1
	Inline skates*	36.0 ± 17.7	32.6 ± 14.7	34.3 ± 16.4
	Roller shoes	44.2 ± 37.0	43.6 ± 45.7	44.0 ± 40.7
	Skateboard*	33.9 ± 20.0	31.2 ± 16.2	32.6 ± 18.2
	Kick scooter*	26.3 ± 13.1	24.9 ± 11.9	25.7 ± 12.6
	Picnic mat*	76.5 ± 57.7	56.0 ± 49.2	71.7 ± 56.5
Stationery	Oil pastel*	22.7 ± 14.0	21.3 ± 10.5	22.1 ± 12.5
	Colored pencil*	19.8 ± 10.3	17.3 ± 9.4	18.6 ± 10.0
	Paint supplies*	23.6 ± 14.6	21.0 ± 11.5	22.7 ± 13.7
	Workbook*	24.8 ± 12.2	23.8 ± 11.7	24.3 ± 11.9
	Sticker/sticker book*	13.8 ± 8.5	11.6 ± 7.9	12.8 ± 8.3
	Notebook*^a^	27.0 ± 25.8	26.1 ± 21.2	26.5 ± 23.7
	Ballpoint pen*^a^	16.1 ± 18.4	14.0 ± 12.7	15.0 ± 15.7
	Penci*^a^	29.7 ± 29.3	28.1 ± 24.9	28.9 ± 27.3
	Marker pen*^a^	16.4 ± 16.8	11.4 ± 7.7	14.2 ± 13.8
	Eraser	NA	NA	NA
	Correction tape/fluid	NA	NA	NA
	Glue	NA	NA	NA
	Adhesive	NA	NA	NA
	Scissors	NA	NA	NA

*AM* arithmetic mean, *STD* standard deviation.

*Products with significant seasonal differences in the *t* test (*p* ≤ 0.001).

^a^min/day.

## Discussion

### Survey design

This study established a Korean national representative exposure factor database for children’s product usage patterns using proportional quota sampling. The target population for a single survey was 10,000, and substantial respondents for the smallest population area and rarely used products were considered. Among the 10,000 respondents of 300 quotas in a single survey, the smallest quota was 7 for children aged 0 years in the county area of Gyeonggi. Conversely, the largest was 125 for boys 7–9 years old in the city area of Gyeonggi. The product with the lowest use rate (skateboard in the summer survey) had 570 respondents in a single survey. These sampling methods and sample sizes enabled the collection of representative exposure factors for children’s products.

### Age group determination

Children’s age group division is an important component in the management of children’s products. The main consideration of this study was the key living space Korean children mainly stay or perform activities. For 0–2 years, their major living space was their own home. For 3–6 years, it was their own home and childcare facilities, such as daycare centers and kindergartens. For 7–12 years, it was their own homes and elementary schools. The age quotas of the preschool children were divided into two-year-old units except for babies aged 0–12 months. Elementary school children’s age quotas were divided into three-year-old units by grade level as lower- and higher-grade elementary school children might differ in lifestyle and product usage patterns. Finally, exposure factors were presented for the four age groups: infants aged 0–2, toddlers aged 3–6, lower-grade elementary children aged 7–9, and higher-grade elementary children aged 10–12.

In the Special Act on the Safety of Products for Children, Toy Safety Directive 2009/48/EC, and Safety Standard Mandating ASTM F963 for Toys of Korea, the EU, and the U.S., respectively, children’s products for children under 3 years (36 months) were more strictly regulated than products for 4–12 years. The age groups varied depending on specific considerations. The age group recommendations for monitoring and assessing childhood exposures considering behavioral physiological changes were in subgroups of <12 months, 1, 2, 3–5, 6–10, and 11–15 years [34]. In consideration of the consumer product skill characteristics, play behaviors, and interest the age groups of children were divided into subgroups of <12 months, 12–18 months, 19–23 months, 2 years, 3 years, 4–5 years, 6–8 years, and 9–12 years [35].

The national exposure factor handbooks in the US [21], China [36], and Korea [26] reflect these considerations and provide exposure factors for each parameter by children’s age group. In the Korean handbook, children’s body weight and total body surface area were classified according to three-month units before 12 months, and one-year-old unit. The body part surface areas of children aged 0–12 months, 1–2, 3–6, 7–9, 10–12, 13–15, and 16–18 years were also provided. The exposure factors of the children’s product usage patterns obtained in this study were matched with the physiological exposure factors in the Korean handbook and could be used for the management of children’s products in risk assessments.

### Comparison of exposure factors

The exposure factors from the nationwide survey were used for a more refined exposure assessment. In the case of products that can be compared directly, default values of the RIVM toys fact sheet [24] for ballpoint pen (1 event/day, 30 min/event) and marker pen (4 event/week, 30 min/event) was much higher than values observed in this study for ballpoint pen (overall 0.40 ± 0.24 event/day, 15.0 ± 15.7 min/event) and marker pen (overall 0.31 ± 0.19 event/day, 14.2 ± 13.8 min/event). The default values of the toy fact sheet derived from the worst-case assumption by a single value had a low quality factor from the RIVM’s quality grading of data reliability [22, 25] for quantitative exposure estimation. In contrast, the exposure factors obtained from the national representative survey in this study had a high quality factor, indicating reliability of the estimation.

The data of products that have undergone risk assessment studies were used for the comparison of exposure factors used for exposure estimation. Several studies on diapers and wet wipes related to skin contact, baby bottles related to oral intake, and teethers and pacifiers related to mouthing activity have been conducted for infants under 36 months. Product usage pattern information about sporting goods for children has rarely been reported. For stationery, usage pattern information of school children was reported for some arts and craft materials.

The frequency of disposable diaper use obtained from the survey [37] was comparable to the results of this study. The mean ranged from 0.3 in India to 5.9 in the U.S., which was slightly lower than the results of this study (overall 6.05 ± 2.87 event/day). The 95th percentile in Japan and the U.S. was 10, the same as this study. The use rate of wet wipes as a baby care product was 87%, and the use frequency was 3 times per day [38] for parents of children aged younger than 3 years in the U.S. In this study, Korean children used wet wipes more often, with a use rate of 96.5% and a mean use frequency of 6.63 ± 4.24 event/day in aged 0–2 years.

The daily bottle-feeding frequency reported to be in the range of 0–7.5 times and mean of 2.3 ± 1.8 times in children aged 12 months [39] was comparable to the use frequency of baby bottles (overall 3.75 ± 1.91 event/day) in Korean children aged 0–2 years of this study. In the United Kingdom, the daily pacifier use duration of children aged 24–71 months was 3.6 h/day [40]. In this study, the average daily pacifier use duration derived from the mean values of use frequency (overall 2.80 ± 1.95 event/day) and use duration (overall 17.58 ± 11.60 min/event) of Korean children aged 0–2 years was 0.82 h/day. For teethers, a mouthing time of 23–27 min/h for soft plastic articles [11] was used to exposure estimation, and one-time exposure (exposure frequency, EF = 1) assumption [41] was used for risk assessment.

The use rates of drawing materials for preschoolers obtained from a survey in Lithuania [42] were 80.1%, 47.9%, and 38.0% for colored pencils, watercolors, and chalks, respectively. In the U.S., 20% of children used markers for at least 30 min at a time [43], and the school children’s mean use durations for crayons, glues, and pencils were 5.9, 3.97, and 5.83 h/month, respectively [44]. Korean children participated less in drawing activities than U.S. children. In this study, children aged 7–12 years old used oil pastels, colored pencils, marker pen, and pencils at an average of 3.55, 3.74, 7.87, and 18.38 h/month, respectively. The monthly use durations of oil pastels and colored pencils were derived from the mean value of use frequency (event/day) and use duration (min/event). In this study on some stationery products, such as notebooks, pencils, ballpoint pens, and marker pens, directly investigated the daily use duration (min/day); hence the use frequency (event/day) of these products was difficult to specify.

The exposure factor results presented in this study can be used as input parameters for refined exposure assessment considering children’s sex, age, and season. Among sex, age, and seasonal differences, the most obvious difference was that among age groups. Sex differences were rarely observed in infants. The seasonal differences in use rate and time may be related to outdoor activities as the products with a 20% higher use rate in summer were beach balls, picnic mats, swimming goggles, lunch boxes, baby bottles, and bubble-making toys. In the case of beach balls and picnic mats, their use duration in summer was 9.1 and 20.5 min/event higher than that in winter. The remaining four products had similar use durations in summer and winter. In addition, the average differences in use frequency for these products were small, ranging only 0.02–0.20 event/day, despite the significant differences. These results show that the seasonal difference was mainly affected by the use or non-use of a specific product, and that the quantitative exposure factors as input parameters of exposure estimation were less related.

The exposure factor was investigated by considering the product characteristics and the exposure algorithm. The determination of the single event and use duration (min/event) of some stationery products, such as erasers, correction tape/fluid, glue, adhesives, and scissors were difficult. For wet wipes, the use amount (ea./event or g/ea.) was more important for quantitative exposure estimation than use duration. Food containers, such as food trays, lunch boxes, and water bottles, had difficulty defining contact event with the human body. The concentration characteristics by material, such as the migration level, would be more important for risk assessment than product usage pattern characteristics.

### Limitations

The exposure factor survey in this study was conducted twice, once in both summer and winter; however, the same population was not surveyed. As the period between the summer and winter surveys was eight months, some respondents had to change age groups due to the growth of the children. The derived seasonal difference might be caused by the difference in seasons and between respondents. Also, the quota sampling method had a potential for selection bias. It was not accessible to those who are not initial 40,000 panels. The sampling error cannot be assessed since the sample was not randomized. Nevertheless, the 300 quotas which considering the sex, age, and regional distribution children are sufficiently representing Korean children’s population. Deriving nationally representative results was still possible.

The survey questionnaire was developed for the children’s parents. Although elementary school children responded with their parents, parents’ responses might not have accurately described their children’s product usage patterns. This was a fundamental limitation of the survey method and not of the observation method. However, face-to-face interview surveys are more suitable than observations in investigating the usage patterns of children’s products for various categories due to resource and financial restrictions. There was no verification about children’s behavior with products at childcare facilities or school. Further surveys for children’s products will be able to select additional products based on the actual observation in children’s activity space and interview with the kindergarten or school teacher.

## Conclusions

This study determined the exposure factors of 57 children’s products through twice face-to-face interviews with each 10,000 households in Korea and constructed a nationally representative exposure factor database. The use rate, use frequency, and use duration of the children’s products revealed sex, age, and seasonal differences. According to the characteristics of each group, these exposure factors can be used to accurately refine exposure assessments and set safety guidelines for children’s products.

## Supplementary Information

Supplementary Information

## Data Availability

The data presented in this study are available on request from the corresponding author. The survey questionnaires are available to download from https://library.me.go.kr/#/search/detail/5704256 (in Korean).
