# Supplementary material for: Development of nationally representative exposure factor database for children’s products in Korea
Source: J Expo Sci Environ Epidemiol. 2024 Feb 29;34(6):1054–63. doi: 10.1038/s41370-024-00654-1 (PMC11618069; doi:10.1038/s41370-024-00654-1)
Supplement: Supplementary file 1 — Supplementary Information [file 41370_2024_654_MOESM1_ESM.docx]

**Supplementary Information**

**Development of nationally representative exposure factor database
for children’s products in Korea**

Jiyun Shin^1^, Kiyoung Lee^1,2^, Seung Yeol Park^3^, Miyoung Lim^2*^

1 Department of Environmental Health Sciences, Graduate School of Public Health, Seoul National University, Seoul, Republic of Korea

2 Institute of Health and Environment, Seoul National University, Seoul, Republic of Korea

3 KSTAT Research, Seoul, Republic of Korea

* Corresponding author: [me02me0@snu.ac.kr](mailto:me02me0@snu.ac.kr) (M. Lim).

**Table S1.** Use rates (%) of children’s product by gender for each season’s survey.

**Table S2.** Use rates (%) of children’s product by age groups for each season’s survey.

**Table S3.** Use frequency (AM ± STD, event/day) of children’s product by gender for each season’s survey.

**Table S4.** Use frequency (AM ± STD, event/day) of children’s product by age groups for each season’s survey.

**Table S5.** Percentiles for use frequency (event/day) of children’s product by season and gender.

**Table S6.** Percentiles for use frequency (event/day) of children’s product by age group.

**Table S7.** Use durations (AM ± STD, min/event) of children’s product by gender for each season’s survey.

**Table S8.** Use durations (AM ± STD, min/event) of children’s product by age groups for each season’s survey.

**Table S9.** Percentiles for use duration (min/event) of children’s product by season and gender.

**Table S10.** Percentiles for use duration (min/event) of children’s product by season and gender.

**Table S1.** Use rates (%) of children’s product by gender for each season’s survey.

| Category | Product | Summer |  | Winter |  | Overall |  |  |
| --- | --- | --- | --- | --- | --- | --- | --- | --- |
|  |  | Boy | Girl | Boy | Girl | Boy | Girl | Significance |
| Baby product | Self-righting toy | 48.5 | 48.2 | 47.3 | 50.8 | 47.9 | 49.5 | ns |
|  | Baby rattle | 45.8 | 42.5 | 38.8 | 40.0 | 42.3 | 41.2 | * |
|  | Squeaky toy | 75.1 | 73.0 | 78.0 | 71.2 | 76.6 | 72.1 | ns |
|  | Tactile toy | 73.1 | 75.7 | 72.7 | 75.7 | 72.9 | 75.7 | ns |
|  | Baby mobile toy | 23.9 | 22.9 | 15.9 | 14.5 | 19.9 | 18.7 | ns |
|  | Teether | 44.3 | 42.7 | 36.2 | 37.6 | 40.3 | 40.2 | ns |
|  | Pacifier | 53.6 | 52.4 | 41.7 | 43.5 | 47.6 | 47.9 | ns |
|  | Baby bouncer | 44.4 | 45.6 | 24.9 | 26.3 | 34.6 | 36.0 | ns |
|  | Baby walker | 34.8 | 32.2 | 22.0 | 21.7 | 28.4 | 26.9 | ns |
|  | Diaper | 86.9 | 86.1 | 83.1 | 82.4 | 84.9 | 84.3 | ns |
|  | Teeth wipes | 34.1 | 34.0 | 29.2 | 30.3 | 31.7 | 32.1 | ns |
|  | Baby bottle | 77.8 | 73.7 | 51.7 | 51.6 | 64.8 | 62.6 | ns |
|  | Baby playpen | 42.5 | 42.1 | 25.1 | 21.7 | 33.8 | 31.9 | *** |
| Toy | Play sand | 28.3 | 33.4 | 23.3 | 28.0 | 25.8 | 30.7 | *** |
|  | Bubble-making toy | 60.6 | 60.3 | 39.7 | 40.4 | 50.1 | 50.3 | ns |
|  | Kid's car | 52.5 | 47.6 | 43.1 | 38.3 | 47.8 | 42.9 | *** |
|  | Kid's bike | 53.9 | 52.7 | 41.6 | 42.2 | 47.7 | 47.5 | ns |
|  | Card game | 69.1 | 53.7 | 61.8 | 40.3 | 65.4 | 47.0 | *** |
|  | Board game | 55.7 | 53.2 | 54.6 | 46.7 | 55.1 | 49.9 | *** |
|  | Electronic game | 31.8 | 23.5 | 37.3 | 24.3 | 34.5 | 23.9 | *** |
|  | Toy audio player | 46.8 | 48.3 | 36.6 | 39.0 | 41.7 | 43.7 | ** |
|  | Toy video player | 36.2 | 38.5 | 35.0 | 37.6 | 35.6 | 38.0 | *** |
|  | Beach ball | 69.1 | 66.5 | 23.0 | 20.5 | 46.0 | 43.5 | *** |
|  | Swimming goggles | 63.2 | 62.8 | 18.8 | 16.2 | 41.0 | 39.5 | ns |
|  | Bath toy | 74.1 | 74.6 | 56.9 | 53.5 | 65.5 | 64.0 | ns |
| Daily product | Wet wipes | 96.6 | 97.6 | 95.2 | 96.5 | 95.9 | 97.1 | *** |
|  | Toothbrush | 97.0 | 97.1 | 96.3 | 96.7 | 96.7 | 96.9 | ns |
|  | Cotton swab | 76.8 | 76.5 | 85.9 | 86.7 | 81.3 | 81.6 | ns |
|  | Towel | 99.8 | 99.6 | 99.1 | 99.1 | 99.4 | 99.4 | ns |
|  | Handkerchief | 46.5 | 48.4 | 43.0 | 49.2 | 44.7 | 48.8 | *** |
|  | Food tray | 83.9 | 84.6 | 82.2 | 82.2 | 83.0 | 83.4 | ns |
|  | Lunch box | 56.5 | 57.5 | 31.6 | 34.3 | 44.0 | 45.9 | ** |
|  | Water bottle | 92.9 | 92.9 | 81.8 | 84.9 | 87.3 | 88.9 | *** |
|  | Car seat | 80.6 | 80.0 | 71.4 | 69.3 | 45.3 | 44.7 | ns |
|  | Kid's chair | 53.0 | 51.0 | 42.6 | 39.6 | 28.5 | 27.1 | ** |
| Sporting goods | Ball | 80.2 | 64.0 | 72.0 | 44.7 | 76.1 | 54.4 | *** |
|  | Gloves | 26.6 | 10.4 | 28.6 | 2.8 | 27.6 | 6.6 | *** |
|  | Bicycle | 64.7 | 58.1 | 62.2 | 44.4 | 63.5 | 51.2 | *** |
|  | Inline skates | 26.0 | 27.2 | 25.1 | 27.0 | 25.6 | 27.1 | * |
|  | Roller shoes | 15.4 | 16.9 | 9.0 | 12.8 | 12.2 | 14.8 | *** |
|  | Skateboard | 9.0 | 6.6 | 11.3 | 4.8 | 10.1 | 5.7 | *** |
|  | Kick scooter | 58.3 | 58.9 | 38.9 | 45.0 | 48.6 | 52.0 | *** |
|  | Picnic mat | 64.4 | 67.2 | 19.2 | 20.9 | 41.8 | 44.0 | ** |
| Stationery | Oil pastel | 77.1 | 76.6 | 65.3 | 67.0 | 71.2 | 71.8 | ns |
|  | Colored pencil | 85.1 | 85.2 | 73.7 | 77.6 | 79.4 | 81.4 | *** |
|  | Paint supplies | 27.9 | 31.4 | 13.7 | 17.7 | 20.8 | 24.5 | *** |
|  | Workbook | 71.6 | 70.7 | 70.9 | 71.3 | 71.2 | 71.0 | ns |
|  | Sticker/sticker book | 59.6 | 67.3 | 50.5 | 60.5 | 55.1 | 63.9 | *** |
|  | Notebook | 93.9 | 93.3 | 90.5 | 91.0 | 92.2 | 92.1 | ns |
|  | Ballpoint pen | 39.4 | 42.6 | 43.3 | 45.4 | 41.3 | 44.0 | *** |
|  | Pencil | 95.9 | 95.7 | 93.6 | 93.0 | 94.8 | 94.3 | ns |
|  | Marker pen | 57.3 | 60.1 | 45.8 | 45.7 | 51.5 | 52.9 | ns |
|  | Eraser | 94.2 | 94.2 | 89.8 | 90.7 | 92.0 | 92.5 | ns |
|  | Correction tape/fluid | 12.0 | 16.6 | 19.7 | 21.0 | 15.9 | 18.8 | *** |
|  | Glue | 91.2 | 92.5 | 76.7 | 78.8 | 83.9 | 85.6 | ** |
|  | Adhesive | 13.2 | 13.0 | 11.5 | 10.5 | 12.4 | 11.7 | ns |
|  | Scissors | 95.6 | 96.4 | 85.1 | 86.3 | 90.3 | 91.4 | ns |

NA: Not available.

ns: p > 0.05, *: p ≤ 0.05, **: p ≤ 0.01, ***: p ≤ 0.001, ****: p ≤ 0.0001.

**Table S2.** Use rates (%) of children’s product by age groups for each season’s survey.

| Category | Product | Summer |  |  |  | Winter |  |  |  | Overall |  |  |  |  |
| --- | --- | --- | --- | --- | --- | --- | --- | --- | --- | --- | --- | --- | --- | --- |
|  |  | 0-2 yrs | 3-6 yrs | 7-9 yrs | 10-12 yrs | 0-2 yrs | 3-6 yrs | 7-9 yrs | 10-12 yrs | 0-2 yrs | 3-6 yrs | 7-9 yrs | 10-12 yrs | Significance |
| Baby product | Self-righting toy | 48.4 | NA | NA | NA | 49.0 | NA | NA | NA | 48.7 | NA | NA | NA | - |
|  | Baby rattle | 44.1 | NA | NA | NA | 39.4 | NA | NA | NA | 41.8 | NA | NA | NA | - |
|  | Squeaky toy | 74.1 | NA | NA | NA | 74.6 | NA | NA | NA | 74.4 | NA | NA | NA | - |
|  | Tactile toy | 74.4 | NA | NA | NA | 74.2 | NA | NA | NA | 74.3 | NA | NA | NA | - |
|  | Baby mobile toy | 23.4 | NA | NA | NA | 15.2 | NA | NA | NA | 19.3 | NA | NA | NA | - |
|  | Teether | 43.5 | NA | NA | NA | 36.9 | NA | NA | NA | 40.2 | NA | NA | NA | - |
|  | Pacifier | 53.0 | NA | NA | NA | 42.6 | NA | NA | NA | 47.8 | NA | NA | NA | - |
|  | Baby bouncer | 45.0 | NA | NA | NA | 25.6 | NA | NA | NA | 35.3 | NA | NA | NA | - |
|  | Baby walker | 33.5 | NA | NA | NA | 21.8 | NA | NA | NA | 27.6 | NA | NA | NA | - |
|  | Diaper | 86.5 | NA | NA | NA | 82.8 | NA | NA | NA | 84.6 | NA | NA | NA | - |
|  | Teeth wipes | 34.0 | NA | NA | NA | 29.7 | NA | NA | NA | 31.9 | NA | NA | NA | - |
|  | Baby bottle | 75.8 | NA | NA | NA | 51.7 | NA | NA | NA | 63.7 | NA | NA | NA | - |
|  | Baby playpen | 42.3 | NA | NA | NA | 23.5 | NA | NA | NA | 32.9 | NA | NA | NA | - |
| Toy | Play sand | 26.4 | 41.2 | 30.9 | 19.5 | 22.6 | 32.5 | 25.7 | 18.2 | 24.5 | 36.8 | 28.3 | 18.9 | *** |
|  | Bubble-making toy | 54.8 | 81.0 | 57.4 | 37.3 | 39.5 | 53.3 | 34.2 | 24.9 | 47.1 | 67.2 | 45.8 | 31.1 | *** |
|  | Kid's car | 55.7 | 45.5 | NA | NA | 61.2 | 24.1 | NA | NA | 58.5 | 34.8 | NA | NA | *** |
|  | Kid's bike | 51.5 | 54.7 | NA | NA | 45.5 | 39.0 | NA | NA | 48.5 | 46.9 | NA | NA | ns |
|  | Card game | NA | 54.9 | 68.1 | 65.7 | NA | 50.0 | 54.7 | 49.7 | NA | 52.5 | 61.4 | 57.7 | *** |
|  | Board game | NA | 39.7 | 61.4 | 71.7 | NA | 41.5 | 54.9 | 61.5 | NA | 40.6 | 58.1 | 66.6 | *** |
|  | Electronic game | NA | 16.2 | 31.9 | 42.3 | NA | 23.7 | 34.5 | 39.2 | NA | 20.0 | 33.2 | 40.8 | *** |
|  | Toy audio player | 91.6 | 56.9 | 14.1 | 7.3 | 77.6 | 41.6 | 10.1 | 6.6 | 84.6 | 49.2 | 12.1 | 7.0 | **** |
|  | Toy video player | 58.1 | 51.9 | 16.1 | 7.1 | 66.6 | 46.1 | 10.4 | 6.2 | 62.3 | 49.0 | 13.2 | 6.7 | **** |
|  | Beach ball | 63.6 | 79.6 | 64.7 | 57.2 | 31.9 | 20.7 | 16.4 | 15.3 | 47.8 | 50.2 | 40.6 | 36.2 | *** |
|  | Swimming goggles | NA | 48.5 | 76.9 | 72.7 | NA | 13.9 | 21.8 | 19.0 | NA | 31.2 | 49.4 | 45.8 | *** |
|  | Bath toy | 93.4 | 58.9 | NA | NA | 75.9 | 38.4 | NA | NA | 84.6 | 48.7 | NA | NA | **** |
| Daily product | Wet wipes | 99.9 | 98.7 | 95.2 | 92.6 | 99.7 | 96.6 | 94.2 | 91.1 | 99.8 | 97.7 | 94.7 | 91.8 | *** |
|  | Toothbrush | 89.6 | 100.0 | 99.4 | 99.9 | 87.1 | 100.0 | 99.9 | 99.9 | 88.3 | 100.0 | 99.6 | 99.9 | **** |
|  | Cotton swab | 82.0 | 74.1 | 72.7 | 77.5 | 92.1 | 85.0 | 82.1 | 85.0 | 87.1 | 79.6 | 77.4 | 81.3 | *** |
|  | Towel | 100.0 | 99.5 | 99.9 | 99.6 | 99.5 | 99.0 | 98.7 | 99.0 | 99.8 | 99.3 | 99.3 | 99.3 | ** |
|  | Handkerchief | 89.9 | 44.1 | 23.4 | 20.5 | 74.0 | 37.5 | 34.1 | 35.1 | 81.9 | 40.8 | 28.7 | 27.8 | **** |
|  | Food tray | 61.9 | 94.8 | 90.7 | 90.2 | 55.4 | 93.0 | 90.6 | 91.7 | 58.6 | 93.9 | 90.6 | 90.9 | **** |
|  | Lunch box | 28.4 | 77.2 | 60.2 | 58.6 | 16.7 | 40.8 | 37.6 | 36.9 | 22.6 | 59.0 | 48.9 | 47.8 | **** |
|  | Water bottle | 92.4 | 95.7 | 90.3 | 91.6 | 84.5 | 87.7 | 79.5 | 78.3 | 88.5 | 91.7 | 84.9 | 84.9 | *** |
|  | Car seat | 97.9 | 66.1 | NA | NA | 92.6 | 52.3 | NA | NA | 95.2 | 59.2 | NA | NA | **** |
|  | Kid's chair | 79.9 | 29.3 | NA | NA | 67.4 | 19.8 | NA | NA | 73.7 | 24.5 | NA | NA | **** |
| Sporting goods | Ball | 67.4 | 78.6 | 69.1 | 71.2 | 55.3 | 59.9 | 60.3 | 59.2 | 61.3 | 69.3 | 64.7 | 65.2 | *** |
|  | Gloves | NA | 9.3 | 23.5 | 29.0 | NA | 8.8 | 19.4 | 24.2 | NA | 9.1 | 21.4 | 26.6 | *** |
|  | Bicycle | NA | 42.3 | 78.8 | 75.7 | NA | 36.3 | 65.5 | 69.4 | NA | 39.3 | 72.1 | 72.5 | **** |
|  | Inline skates | NA | 11.6 | 37.3 | 40.5 | NA | 18.1 | 33.1 | 32.1 | NA | 14.9 | 35.2 | 36.3 | *** |
|  | Roller shoes | NA | 4.2 | 22.2 | 29.7 | NA | 5.0 | 16.3 | 14.9 | NA | 4.6 | 19.3 | 22.3 | *** |
|  | Skateboard | NA | 1.4 | 9.0 | 17.0 | NA | 4.2 | 8.2 | 14.3 | NA | 2.8 | 8.6 | 15.7 | *** |
|  | Kick scooter | NA | 77.1 | 54.1 | 32.9 | NA | 56.8 | 37.3 | 22.3 | NA | 66.9 | 45.7 | 27.6 | **** |
|  | Picnic mat | 64.4 | 75.5 | 63.2 | 54.3 | 17.8 | 22.1 | 19.4 | 20.2 | 41.1 | 48.8 | 41.3 | 37.3 | *** |
| Stationery | Oil pastel | 44.2 | 92.8 | 92.6 | 78.5 | 28.8 | 88.4 | 80.7 | 64.8 | 36.5 | 90.6 | 86.6 | 71.6 | **** |
|  | Colored pencil | 54.3 | 97.9 | 96.9 | 93.4 | 49.6 | 92.3 | 86.3 | 72.2 | 51.9 | 95.1 | 91.6 | 82.8 | **** |
|  | Paint supplies | 16.6 | 32.5 | 36.1 | 35.8 | 4.6 | 18.9 | 19.6 | 20.9 | 10.6 | 25.7 | 27.8 | 28.4 | *** |
|  | Workbook | 16.6 | 85.9 | 94.7 | 95.8 | 23.3 | 80.2 | 95.4 | 95.3 | 20.0 | 83.1 | 95.1 | 95.6 | **** |
|  | Sticker/sticker book | 51.3 | 85.5 | 60.2 | 46.7 | 42.0 | 72.6 | 56.8 | 43.7 | 46.6 | 79.0 | 58.5 | 45.2 | **** |
|  | Notebook | NA | 86.7 | 99.0 | 99.6 | NA | 81.4 | 98.2 | 98.5 | NA | 84.0 | 98.6 | 99.1 | *** |
|  | Ballpoint pen | NA | 28.0 | 39.9 | 63.3 | NA | 23.5 | 48.8 | 73.9 | NA | 25.8 | 44.3 | 68.6 | **** |
|  | Pencil | NA | 92.1 | 98.6 | 99.1 | NA | 86.4 | 98.4 | 99.5 | NA | 89.3 | 98.5 | 99.3 | *** |
|  | Marker pen | NA | 51.5 | 59.5 | 69.5 | NA | 38.7 | 48.7 | 54.1 | NA | 45.1 | 54.1 | 61.8 | *** |
|  | Eraser | NA | 87.3 | 99.6 | 100.0 | NA | 79.9 | 98.8 | 98.6 | NA | 83.6 | 99.2 | 99.3 | *** |
|  | Correction tape/fluid | NA | 1.8 | 13.3 | 35.7 | NA | 3.1 | 24.1 | 44.8 | NA | 2.4 | 18.7 | 40.2 | **** |
|  | Glue | NA | 90.3 | 93.4 | 92.7 | NA | 77.5 | 79.8 | 75.9 | NA | 83.9 | 86.6 | 84.3 | *** |
|  | Adhesive | NA | 4.6 | 18.2 | 21.7 | NA | 5.4 | 14.1 | 17.1 | NA | 5.0 | 16.2 | 19.4 | *** |
|  | Scissors | NA | 94.8 | 96.9 | 97.1 | NA | 81.0 | 90.1 | 89.0 | NA | 87.9 | 93.5 | 93.0 | *** |

NA: Not available.

ns: p > 0.05, *: p ≤ 0.05, **: p ≤ 0.01, ***: p ≤ 0.001, ****: p ≤ 0.0001.

**Table S3.** Use frequency (AM ± STD, event/day) of children’s product by gender for each season’s survey.

| Category | Product | Summer |  | Winter |  | Overall |  |  |
| --- | --- | --- | --- | --- | --- | --- | --- | --- |
|  |  | Boy | Girl | Boy | Girl | Boy | Girl | Significance |
| Baby product | Self-righting toy | 1.59±1.47 | 1.50±1.37 | 1.31±1.35 | 1.31±1.43 | 1.45±1.42 | 1.40±1.40 | ns |
|  | Baby rattle | 1.98±1.98 | 1.96±1.95 | 1.96±1.81 | 1.89±1.86 | 1.97±1.90 | 1.93±1.91 | ns |
|  | Squeaky toy | 1.63±1.51 | 1.62±1.51 | 1.38±1.29 | 1.20±1.19 | 1.50±1.41 | 1.41±1.38 | ns |
|  | Tactile toy | 1.58±1.39 | 1.50±1.39 | 1.36±1.51 | 1.27±1.39 | 1.47±1.45 | 1.38±1.39 | ns |
|  | Baby mobile toy | 1.43±1.18 | 1.69±1.88 | 1.70±1.50 | 1.79±1.57 | 1.54±1.32 | 1.73±1.76 | ns |
|  | Teether | 2.39±1.97 | 2.38±1.84 | 1.83±1.43 | 1.89±1.70 | 2.14±1.77 | 2.15±1.79 | * |
|  | Pacifier | 3.07±2.10 | 2.95±1.96 | 2.54±1.86 | 2.55±1.77 | 2.84±2.01 | 2.77±1.89 | ns |
|  | Baby bouncer | 2.02±1.53 | 2.05±1.44 | 1.36±1.22 | 1.39±1.30 | 1.79±1.46 | 1.81±1.42 | ns |
|  | Baby walker | 2.05±1.79 | 2.14±1.66 | 2.05±1.55 | 2.15±1.60 | 2.05±1.70 | 2.14±1.64 | * |
|  | Diaper | 6.49±2.78 | 6.47±3.01 | 5.58±2.77 | 5.62±2.79 | 6.04±2.81 | 6.05±2.94 | ns |
|  | Teeth wipes | 1.77±0.92 | 1.77±1.05 | 1.36±0.97 | 1.30±0.95 | 1.58±0.97 | 1.55±1.03 | ns |
|  | Baby bottle | 3.92±1.94 | 3.74±1.94 | 3.70±1.83 | 3.56±1.88 | 3.83±1.90 | 3.66±1.92 | ns |
|  | Baby playpen | 1.81±1.48 | 1.76±1.46 | 1.47±1.45 | 1.42±1.37 | 1.68±1.48 | 1.64±1.44 | * |
| Toy | Play sand | 0.13±0.20 | 0.12±0.19 | 0.12±0.13 | 0.12±0.14 | 0.12±0.17 | 0.12±0.17 | ns |
|  | Bubble-making toy | 0.11±0.20 | 0.10±0.15 | 0.08±0.15 | 0.07±0.12 | 0.10±0.18 | 0.09±0.14 | * |
|  | Kid's car | 1.05±1.25 | 0.97±1.18 | 0.88±1.07 | 0.82±0.98 | 0.97±1.17 | 0.90±1.10 | * |
|  | Kid's bike | 0.57±0.57 | 0.53±0.56 | 0.36±0.50 | 0.37±0.47 | 0.48±0.55 | 0.46±0.53 | ns |
|  | Card game | 0.51±0.74 | 0.44±0.67 | 0.41±0.58 | 0.31±0.46 | 0.46±0.67 | 0.38±0.59 | **** |
|  | Board game | 0.25±0.38 | 0.24±0.36 | 0.19±0.26 | 0.18±0.29 | 0.22±0.33 | 0.21±0.33 | ns |
|  | Electronic game | 0.43±0.55 | 0.41±0.57 | 0.37±0.45 | 0.38±0.51 | 0.39±0.50 | 0.39±0.54 | ns |
|  | Toy audio player | 1.01±1.05 | 1.00±1.03 | 0.92±1.02 | 0.90±1.05 | 0.97±1.03 | 0.96±1.04 | ns |
|  | Toy video player | 0.92±0.92 | 0.86±0.90 | 0.74±0.93 | 0.69±0.83 | 0.83±0.93 | 0.78±0.87 | ** |
|  | Beach ball | 0.11±0.26 | 0.10±0.25 | 0.28±0.52 | 0.28±0.52 | 0.15±0.35 | 0.14±0.34 | ns |
|  | Swimming goggles | 0.11±0.24 | 0.12±0.21 | 0.21±0.35 | 0.26±0.52 | 0.13±0.27 | 0.14±0.31 | ns |
|  | Bath toy | 0.64±0.50 | 0.64±0.49 | 0.45±0.50 | 0.46±0.48 | 0.56±0.51 | 0.56±0.49 | ns |
| Daily product | Wet wipes | 4.40±3.77 | 4.52±3.86 | 3.14±2.88 | 3.21±2.88 | 3.77±3.42 | 3.87±3.47 | ns |
|  | Toothbrush | 2.48±0.66 | 2.49±0.64 | 2.38±0.65 | 2.39±0.65 | 2.43±0.66 | 2.44±0.65 | ns |
|  | Cotton swab | 0.39±0.51 | 0.42±0.54 | 0.33±0.42 | 0.33±0.42 | 0.36±0.47 | 0.37±0.48 | ns |
|  | Towel | 2.93±1.39 | 2.91±1.37 | 2.93±1.23 | 2.96±1.24 | 2.93±1.31 | 2.93±1.30 | ns |
|  | Handkerchief | 2.40±2.77 | 2.31±2.60 | 1.70±2.17 | 1.50±2.13 | 2.07±2.52 | 1.90±2.41 | *** |
|  | Food tray | 1.28±0.89 | 1.31±0.95 | 1.11±0.87 | 1.11±0.90 | 1.20±0.89 | 1.21±0.94 | ns |
|  | Lunch box | 0.06±0.22 | 0.06±0.26 | 0.06±0.15 | 0.08±0.24 | 0.06±0.19 | 0.07±0.25 | ns |
|  | Water bottle | 1.83±2.08 | 1.84±2.19 | 1.40±1.65 | 1.32±1.53 | 1.63±1.90 | 1.59±1.92 | ns |
|  | Car seat | 0.51±0.60 | 0.51±0.62 | 0.35±0.45 | 0.36±0.46 | 0.43±0.54 | 0.44±0.55 | ns |
|  | Kid's chair | 1.91±1.34 | 1.90±1.20 | 1.89±1.19 | 1.91±1.18 | 1.90±1.28 | 1.90±1.19 | ns |
| Sporting goods | Ball | 0.40±0.69 | 0.37±0.75 | 0.37±0.67 | 0.31±0.60 | 0.39±0.68 | 0.35±0.69 | *** |
|  | Gloves | 0.25±0.50 | 0.25±0.46 | 0.14±0.15 | 0.19±0.25 | 0.19±0.37 | 0.24±0.42 | * |
|  | Bicycle | 0.45±0.55 | 0.37±0.44 | 0.33±0.44 | 0.24±0.33 | 0.39±0.50 | 0.32±0.40 | **** |
|  | Inline skates | 0.21±0.25 | 0.21±0.23 | 0.17±0.29 | 0.16±0.22 | 0.19±0.27 | 0.19±0.22 | ns |
|  | Roller shoes | 0.58±0.91 | 0.47±0.66 | 0.44±0.75 | 0.46±0.74 | 0.53±0.86 | 0.47±0.69 | ns |
|  | Skateboard | 0.20±0.28 | 0.20±0.30 | 0.13±0.18 | 0.12±0.15 | 0.16±0.23 | 0.17±0.25 | ns |
|  | Kick scooter | 0.40±0.44 | 0.38±0.43 | 0.31±0.46 | 0.26±0.38 | 0.37±0.45 | 0.33±0.42 | **** |
|  | Picnic mat | 0.05±0.10 | 0.06±0.15 | 0.07±0.22 | 0.08±0.23 | 0.06±0.14 | 0.07±0.17 | * |
| Stationery | Oil pastel | 0.37±0.42 | 0.39±0.43 | 0.32±0.49 | 0.31±0.43 | 0.35±0.45 | 0.35±0.43 | ns |
|  | Colored pencil | 0.50±0.63 | 0.53±0.67 | 0.39±0.50 | 0.41±0.56 | 0.45±0.58 | 0.47±0.62 | * |
|  | Paint supplies | 0.27±0.41 | 0.27±0.37 | 0.20±0.33 | 0.19±0.28 | 0.25±0.39 | 0.24±0.34 | ns |
|  | Workbook | 0.74±0.82 | 0.71±0.73 | 0.67±0.74 | 0.69±0.83 | 0.70±0.78 | 0.70±0.78 | ns |
|  | Sticker/sticker book | 0.38±0.47 | 0.41±0.52 | 0.27±0.38 | 0.31±0.42 | 0.33±0.44 | 0.36±0.48 | **** |
|  | Notebook | NA | NA | NA | NA | NA | NA | - |
|  | Ballpoint pen | NA | NA | NA | NA | NA | NA | - |
|  | Pencil | NA | NA | NA | NA | NA | NA | - |
|  | Marker pen | NA | NA | NA | NA | NA | NA | - |
|  | Eraser | 5.9±8.8 | 6.1±10.9 | 5.1±4.6 | 5.4±5.3 | 5.5±7.0 | 5.8±8.6 | * |
|  | Correction tape/fluid | 2.7±2.2 | 2.9±3.6 | 2.8±1.6 | 2.8±1.6 | 2.7±1.9 | 2.8±2.6 | ns |
|  | Glue | 3.3±3.6 | 3.6±3.7 | 3.6±3.0 | 3.7±3.0 | 3.4±3.3 | 3.6±3.4 | *** |
|  | Adhesive | 2.3±2.7 | 2.4±2.7 | 2.9±3.2 | 2.9±2.9 | 2.6±3.0 | 2.6±2.8 | ns |
|  | Scissors | 3.6±4.5 | 3.8±4.3 | 3.7±3.3 | 3.8±3.5 | 3.6±3.9 | 3.8±3.9 | * |

NA: Not available.

ns: p > 0.05, *: p ≤ 0.05, **: p ≤ 0.01, ***: p ≤ 0.001, ****: p ≤ 0.0001.

**Table S4.** Use frequency (AM ± STD, event/day) of children’s product by age groups for each season’s survey.

| Category | Product | Summer |  |  |  | Winter |  |  |  | Overall |  |  |  |  |
| --- | --- | --- | --- | --- | --- | --- | --- | --- | --- | --- | --- | --- | --- | --- |
|  |  | 0-2 yrs | 3-6 yrs | 7-9 yrs | 10-12 yrs | 0-2 yrs | 3-6 yrs | 7-9 yrs | 10-12 yrs | 0-2 yrs | 3-6 yrs | 7-9 yrs | 10-12 yrs | Significance |
| Baby product | Self-righting toy | 1.55±1.42 | NA | NA | NA | 1.31±1.39 | NA | NA | NA | 1.43±1.41 | NA | NA | NA | - |
|  | Baby rattle | 1.97±1.96 | NA | NA | NA | 1.93±1.83 | NA | NA | NA | 1.95±1.90 | NA | NA | NA | - |
|  | Squeaky toy | 1.63±1.51 | NA | NA | NA | 1.29±1.25 | NA | NA | NA | 1.46±1.39 | NA | NA | NA | - |
|  | Tactile toy | 1.54±1.39 | NA | NA | NA | 1.31±1.45 | NA | NA | NA | 1.43±1.42 | NA | NA | NA | - |
|  | Baby mobile toy | 1.55±1.56 | NA | NA | NA | 1.74±1.53 | NA | NA | NA | 1.63±1.55 | NA | NA | NA | - |
|  | Teether | 2.39±1.91 | NA | NA | NA | 1.86±1.57 | NA | NA | NA | 2.15±1.78 | NA | NA | NA | - |
|  | Pacifier | 3.01±2.03 | NA | NA | NA | 2.54±1.81 | NA | NA | NA | 2.80±1.95 | NA | NA | NA | - |
|  | Baby bouncer | 2.04±1.48 | NA | NA | NA | 1.38±1.26 | NA | NA | NA | 1.80±1.44 | NA | NA | NA | - |
|  | Baby walker | 2.09±1.73 | NA | NA | NA | 2.10±1.58 | NA | NA | NA | 2.10±1.67 | NA | NA | NA | - |
|  | Diaper | 6.48±2.90 | NA | NA | NA | 5.60±2.78 | NA | NA | NA | 6.05±2.87 | NA | NA | NA | - |
|  | Teeth wipes | 1.77±0.99 | NA | NA | NA | 1.33±0.96 | NA | NA | NA | 1.57±1.00 | NA | NA | NA | - |
|  | Baby bottle | 3.83±1.94 | NA | NA | NA | 3.63±1.86 | NA | NA | NA | 3.75±1.91 | NA | NA | NA | - |
|  | Baby playpen | 1.79±1.47 | NA | NA | NA | 1.45±1.41 | NA | NA | NA | 1.66±1.46 | NA | NA | NA | - |
| Toy | Play sand | 0.06±0.12 | 0.14±0.19 | 0.16±0.25 | 0.12±0.18 | 0.17±0.18 | 0.10±0.11 | 0.11±0.10 | 0.10±0.11 | 0.11±0.16 | 0.12±0.16 | 0.14±0.20 | 0.11±0.15 | **** |
|  | Bubble-making toy | 0.09±0.17 | 0.10±0.16 | 0.13±0.25 | 0.07±0.09 | 0.11±0.16 | 0.07±0.13 | 0.06±0.14 | 0.05±0.07 | 0.10±0.17 | 0.09±0.15 | 0.11±0.22 | 0.06±0.08 | **** |
|  | Kid's car | 1.27±1.41 | 0.76±0.91 | NA | NA | 0.94±1.04 | 0.67±0.97 | NA | NA | 1.10±1.24 | 0.73±0.93 | NA | NA | **** |
|  | Kid's bike | 0.55±0.50 | 0.55±0.60 | NA | NA | 0.39±0.55 | 0.34±0.42 | NA | NA | 0.48±0.53 | 0.46±0.54 | NA | NA | ns |
|  | Card game | NA | 0.46±0.60 | 0.49±0.67 | 0.51±0.88 | NA | 0.43±0.60 | 0.35±0.48 | 0.30±0.47 | NA | 0.44±0.60 | 0.43±0.60 | 0.42±0.74 | ns |
|  | Board game | NA | 0.22±0.39 | 0.26±0.37 | 0.25±0.35 | NA | 0.20±0.30 | 0.18±0.26 | 0.17±0.25 | NA | 0.21±0.35 | 0.22±0.33 | 0.21±0.31 | ns |
|  | Electronic game | NA | 0.37±0.45 | 0.51±0.68 | 0.39±0.51 | NA | 0.42±0.57 | 0.34±0.40 | 0.35±0.43 | NA | 0.40±0.52 | 0.42±0.56 | 0.37±0.48 | ** |
|  | Toy audio player | 1.29±1.16 | 0.77±0.82 | 0.43±0.58 | 0.41±0.60 | 1.19±1.17 | 0.64±0.74 | 0.36±0.46 | 0.27±0.33 | 1.24±1.16 | 0.72±0.79 | 0.40±0.53 | 0.34±0.50 | **** |
|  | Toy video player | 1.17±1.04 | 0.78±0.78 | 0.35±0.46 | 0.38±0.52 | 0.96±1.02 | 0.53±0.68 | 0.29±0.39 | 0.20±0.26 | 1.06±1.03 | 0.66±0.74 | 0.32±0.43 | 0.30±0.43 | **** |
|  | Beach ball | 0.21±0.40 | 0.08±0.22 | 0.06±0.11 | 0.05±0.08 | 0.47±0.71 | 0.19±0.35 | 0.11±0.16 | 0.12±0.19 | 0.30±0.54 | 0.10±0.26 | 0.07±0.12 | 0.07±0.12 | **** |
|  | Swimming goggles | NA | 0.08±0.19 | 0.14±0.27 | 0.11±0.21 | NA | 0.18±0.44 | 0.27±0.47 | 0.25±0.38 | NA | 0.10±0.27 | 0.17±0.33 | 0.14±0.26 | **** |
|  | Bath toy | 0.77±0.36 | 0.47±0.59 | NA | NA | 0.54±0.55 | 0.32±0.34 | NA | NA | 0.67±0.47 | 0.41±0.51 | NA | NA | **** |
| Daily product | Wet wipes | 8.03±4.52 | 3.88±2.53 | 2.50±2.18 | 2.39±1.85 | 5.23±3.42 | 3.00±2.45 | 1.90±1.79 | 1.80±1.75 | 6.63±4.24 | 3.45±2.53 | 2.20±2.02 | 2.10±1.82 | **** |
|  | Toothbrush | 2.21±0.71 | 2.58±0.59 | 2.56±0.61 | 2.56±0.61 | 2.10±0.73 | 2.46±0.61 | 2.50±0.61 | 2.49±0.56 | 2.16±0.72 | 2.52±0.60 | 2.53±0.61 | 2.52±0.59 | **** |
|  | Cotton swab | 0.49±0.57 | 0.35±0.50 | 0.40±0.52 | 0.37±0.48 | 0.47±0.52 | 0.32±0.40 | 0.24±0.33 | 0.23±0.30 | 0.48±0.54 | 0.34±0.45 | 0.32±0.44 | 0.30±0.40 | **** |
|  | Towel | 2.84±1.63 | 2.89±1.27 | 3.06±1.29 | 2.94±1.24 | 2.97±1.40 | 2.95±1.23 | 2.92±1.13 | 2.92±1.10 | 2.90±1.52 | 2.92±1.25 | 2.99±1.22 | 2.93±1.17 | ** |
|  | Handkerchief | 3.56±3.10 | 1.35±1.42 | 0.78±1.05 | 0.69±0.85 | 2.65±2.66 | 0.83±1.13 | 0.76±1.09 | 0.77±1.14 | 3.15±2.94 | 1.11±1.33 | 0.76±1.07 | 0.74±1.05 | **** |
|  | Food tray | 1.61±0.93 | 1.43±0.92 | 1.05±0.83 | 1.05±0.88 | 1.42±0.97 | 1.17±0.93 | 0.96±0.81 | 0.89±0.72 | 1.52±0.95 | 1.30±0.93 | 1.00±0.82 | 0.97±0.81 | **** |
|  | Lunch box | 0.06±0.13 | 0.08±0.33 | 0.05±0.10 | 0.05±0.11 | 0.13±0.33 | 0.08±0.22 | 0.05±0.09 | 0.05±0.10 | 0.08±0.23 | 0.08±0.30 | 0.05±0.10 | 0.05±0.11 | **** |
|  | Water bottle | 2.97±2.61 | 1.87±2.12 | 1.03±1.25 | 1.04±1.25 | 2.08±1.90 | 1.31±1.57 | 0.90±1.13 | 0.89±1.11 | 2.54±2.34 | 1.60±1.90 | 0.97±1.20 | 0.97±1.19 | **** |
|  | Car seat | 0.50±0.60 | 0.52±0.61 | NA | NA | 0.37±0.48 | 0.33±0.43 | NA | NA | 0.44±0.55 | 0.44±0.55 | NA | NA | ns |
|  | Kid's chair | 2.08±1.38 | 1.53±0.90 | NA | NA | 2.05±1.19 | 1.47±1.06 | NA | NA | 2.06±1.30 | 1.51±0.97 | NA | NA | **** |
| Sporting goods | Ball | 0.61±1.23 | 0.29±0.37 | 0.33±0.45 | 0.33±0.38 | 0.60±1.08 | 0.29±0.45 | 0.23±0.27 | 0.25±0.31 | 0.60±1.16 | 0.29±0.40 | 0.28±0.38 | 0.29±0.36 | **** |
|  | Gloves | NA | 0.15±0.17 | 0.26±0.59 | 0.29±0.50 | NA | 0.16±0.18 | 0.15±0.16 | 0.14±0.16 | NA | 0.15±0.17 | 0.21±0.45 | 0.22±0.39 | ** |
|  | Bicycle | NA | 0.36±0.43 | 0.44±0.54 | 0.43±0.53 | NA | 0.28±0.38 | 0.30±0.42 | 0.30±0.40 | NA | 0.33±0.41 | 0.38±0.50 | 0.37±0.47 | **** |
|  | Inline skates | NA | 0.20±0.18 | 0.23±0.29 | 0.20±0.21 | NA | 0.16±0.21 | 0.17±0.22 | 0.17±0.32 | NA | 0.17±0.20 | 0.20±0.26 | 0.19±0.26 | * |
|  | Roller shoes | NA | 0.36±0.53 | 0.49±0.73 | 0.59±0.87 | NA | 0.40±0.51 | 0.41±0.73 | 0.53±0.85 | NA | 0.38±0.52 | 0.46±0.73 | 0.57±0.87 | **** |
|  | Skateboard | NA | 0.12±0.09 | 0.21±0.36 | 0.21±0.26 | NA | 0.13±0.11 | 0.12±0.16 | 0.13±0.19 | NA | 0.13±0.10 | 0.17±0.29 | 0.17±0.23 | ns |
|  | Kick scooter | NA | 0.41±0.40 | 0.35±0.42 | 0.38±0.56 | NA | 0.30±0.41 | 0.27±0.40 | 0.26±0.49 | NA | 0.36±0.41 | 0.32±0.42 | 0.33±0.54 | **** |
|  | Picnic mat | 0.06±0.15 | 0.06±0.11 | 0.06±0.13 | 0.05±0.11 | 0.13±0.36 | 0.05±0.11 | 0.07±0.21 | 0.07±0.18 | 0.08±0.21 | 0.06±0.11 | 0.06±0.15 | 0.06±0.13 | **** |
| Stationery | Oil pastel | 0.37±0.42 | 0.47±0.45 | 0.34±0.42 | 0.27±0.37 | 0.43±0.62 | 0.36±0.43 | 0.25±0.45 | 0.22±0.36 | 0.39±0.51 | 0.41±0.44 | 0.30±0.44 | 0.24±0.37 | **** |
|  | Colored pencil | 0.54±0.70 | 0.65±0.75 | 0.45±0.54 | 0.34±0.47 | 0.48±0.52 | 0.48±0.62 | 0.31±0.47 | 0.25±0.31 | 0.51±0.62 | 0.57±0.69 | 0.38±0.51 | 0.30±0.41 | **** |
|  | Paint supplies | 0.36±0.44 | 0.29±0.37 | 0.26±0.44 | 0.20±0.31 | 0.32±0.41 | 0.22±0.32 | 0.18±0.24 | 0.15±0.29 | 0.35±0.43 | 0.27±0.35 | 0.23±0.38 | 0.18±0.31 | **** |
|  | Workbook | 0.48±0.80 | 0.62±0.66 | 0.77±0.77 | 0.89±0.89 | 0.50±0.69 | 0.57±0.62 | 0.70±0.79 | 0.86±0.96 | 0.50±0.74 | 0.60±0.64 | 0.74±0.78 | 0.88±0.93 | **** |
|  | Sticker/sticker book | 0.36±0.41 | 0.44±0.56 | 0.36±0.46 | 0.34±0.45 | 0.34±0.41 | 0.32±0.42 | 0.23±0.36 | 0.23±0.39 | 0.35±0.41 | 0.39±0.51 | 0.30±0.42 | 0.29±0.42 | **** |
|  | Notebook | NA | NA | NA | NA | NA | NA | NA | NA | NA | NA | NA | NA | - |
|  | Ballpoint pen | NA | NA | NA | NA | NA | NA | NA | NA | NA | NA | NA | NA | - |
|  | Pencil | NA | NA | NA | NA | NA | NA | NA | NA | NA | NA | NA | NA | - |
|  | Marker pen | NA | NA | NA | NA | NA | NA | NA | NA | NA | NA | NA | NA | - |
|  | Eraser | NA | 4.9±4.3 | 6.8±15.6 | 6.7±8.0 | NA | 5.0±4.0 | 5.6±5.8 | 5.5±5.0 | NA | 4.9±4.2 | 6.2±11.8 | 6.1±6.7 | **** |
|  | Correction tape/fluid | NA | 2.2±3.5 | 2.7±2.4 | 2.9±3.3 | NA | 2.4±1.4 | 2.7±1.5 | 2.8±1.6 | NA | 2.3±2.4 | 2.7±1.9 | 2.8±2.5 | * |
|  | Glue | NA | 3.6±3.5 | 3.4±3.9 | 3.2±3.6 | NA | 3.8±3.3 | 3.5±2.8 | 3.4±2.7 | NA | 3.7±3.4 | 3.4±3.4 | 3.3±3.3 | **** |
|  | Adhesive | NA | 3.1±4.0 | 2.1±2.0 | 2.3±2.6 | NA | 3.3±4.1 | 2.9±2.9 | 2.7±2.5 | NA | 3.2±4.1 | 2.5±2.5 | 2.5±2.6 | **** |
|  | Scissors | NA | 3.9±4.7 | 3.5±4.3 | 3.4±3.8 | NA | 4.0±3.5 | 3.7±3.3 | 3.6±3.2 | NA | 3.9±4.2 | 3.6±3.9 | 3.5±3.6 | **** |

NA: Not available.

ns: p > 0.05, *: p ≤ 0.05, **: p ≤ 0.01, ***: p ≤ 0.001, ****: p ≤ 0.0001.

**Table S5.** Percentiles for use frequency (event/day) of children’s product by season and gender.

| Category | Product | Season | | | | | | | | Gender | | | | | | | |
| --- | --- | --- | --- | --- | --- | --- | --- | --- | --- | --- | --- | --- | --- | --- | --- | --- | --- |
|  |  | Summer | | | | Winter | | | | Boy | | | | Girl | | | |
|  |  | 25p | 50p | 75p | 95p | 25p | 50p | 75p | 95p | 25p | 50p | 75p | 95p | 25p | 50p | 75p | 95p |
| Baby product | Self-righting toy | 0.43 | 1 | 2 | 4 | 0.29 | 0.71 | 2 | 4 | 0.29 | 1 | 2 | 4 | 0.29 | 1 | 2 | 4 |
|  | Baby rattle | 0.43 | 2 | 3 | 5 | 0.43 | 2 | 3 | 5 | 0.43 | 2 | 3 | 5 | 0.43 | 2 | 3 | 5 |
|  | Squeaky toy | 0.43 | 1 | 2 | 5 | 0.29 | 1 | 2 | 3 | 0.43 | 1 | 2 | 4 | 0.43 | 1 | 2 | 4 |
|  | Tactile toy | 0.43 | 1 | 2 | 3.80 | 0.43 | 1 | 3 | 4.75 | 0.43 | 1 | 2 | 4 | 0.43 | 1 | 2 | 5 |
|  | Baby mobile toy | 0.43 | 1 | 2 | 4 | 0.29 | 0.71 | 2 | 4 | 0.29 | 1 | 2 | 4 | 0.29 | 1 | 2 | 4 |
|  | Teether | 1 | 2 | 3 | 5 | 0.57 | 2 | 3 | 5 | 1 | 2 | 3 | 5 | 0.71 | 2 | 3 | 5 |
|  | Pacifier | 2 | 3 | 4 | 7.25 | 1 | 2 | 3 | 5 | 2 | 2 | 4 | 6 | 2 | 2 | 4 | 6 |
|  | Baby bouncer | 0.79 | 2 | 3 | 4 | 0.29 | 1 | 2 | 4 | 0.43 | 2 | 3 | 4 | 0.43 | 2 | 3 | 4 |
|  | Baby walker | 0.57 | 2 | 3 | 5 | 1 | 2 | 3 | 5 | 0.57 | 2 | 3 | 5 | 0.71 | 2 | 3 | 5 |
|  | Diaper | 5 | 6 | 8 | 10 | 4 | 6 | 7 | 10 | 5 | 6 | 8 | 10 | 4 | 6 | 8 | 10 |
|  | Teeth wipes | 1 | 2 | 2 | 3 | 0.43 | 1 | 2 | 3 | 1 | 1 | 2 | 3 | 1 | 1 | 2 | 3 |
|  | Baby bottle | 2 | 4 | 5 | 7 | 2 | 4 | 5 | 7 | 2 | 4 | 5 | 7 | 2 | 4 | 5 | 7 |
|  | Baby playpen | 0.43 | 2 | 3 | 5 | 0.29 | 1 | 2 | 4 | 0.43 | 1 | 3 | 4 | 0.43 | 1 | 2 | 4 |
| Toy | Play sand | 0.03 | 0.07 | 0.14 | 0.43 | 0.03 | 0.07 | 0.14 | 0.29 | 0.03 | 0.07 | 0.14 | 0.43 | 0.03 | 0.07 | 0.14 | 0.43 |
|  | Bubble-making toy | 0.02 | 0.03 | 0.14 | 0.43 | 0.01 | 0.03 | 0.07 | 0.29 | 0.02 | 0.03 | 0.10 | 0.29 | 0.02 | 0.03 | 0.10 | 0.29 |
|  | Kid's car | 0.29 | 0.43 | 2 | 3 | 0.14 | 0.43 | 1 | 3 | 0.29 | 0.43 | 1 | 3 | 0.14 | 0.43 | 1 | 3 |
|  | Kid's bike | 0.29 | 0.43 | 0.57 | 2 | 0.10 | 0.29 | 0.43 | 1 | 0.14 | 0.29 | 0.57 | 2 | 0.14 | 0.29 | 0.43 | 2 |
|  | Card game | 0.14 | 0.29 | 0.43 | 2 | 0.10 | 0.14 | 0.43 | 2 | 0.14 | 0.29 | 0.43 | 2 | 0.10 | 0.29 | 0.43 | 1 |
|  | Board game | 0.07 | 0.14 | 0.29 | 0.71 | 0.07 | 0.14 | 0.29 | 0.43 | 0.07 | 0.14 | 0.29 | 0.71 | 0.07 | 0.14 | 0.29 | 0.71 |
|  | Electronic game | 0.13 | 0.29 | 0.43 | 1 | 0.14 | 0.29 | 0.43 | 1 | 0.14 | 0.29 | 0.43 | 1 | 0.13 | 0.29 | 0.43 | 1 |
|  | Toy audio player | 0.29 | 0.57 | 1 | 3 | 0.29 | 0.43 | 1 | 3 | 0.29 | 0.57 | 1 | 3 | 0.29 | 0.57 | 1 | 3 |
|  | Toy video player | 0.29 | 0.57 | 1 | 3 | 0.14 | 0.43 | 1 | 2 | 0.29 | 0.43 | 1 | 2.17 | 0.29 | 0.43 | 1 | 2 |
|  | Beach ball | 0.02 | 0.03 | 0.07 | 0.43 | 0.03 | 0.10 | 0.29 | 1 | 0.02 | 0.03 | 0.14 | 1 | 0.02 | 0.03 | 0.13 | 0.66 |
|  | Swimming goggles | 0.02 | 0.03 | 0.10 | 0.43 | 0.02 | 0.14 | 0.29 | 0.57 | 0.02 | 0.03 | 0.14 | 0.43 | 0.02 | 0.03 | 0.14 | 0.43 |
|  | Bath toy | 0.29 | 0.57 | 1 | 1 | 0.14 | 0.29 | 0.57 | 1 | 0.20 | 0.43 | 1 | 1 | 0.29 | 0.43 | 1 | 1 |
| Daily product | Wet wipes | 2 | 3 | 5 | 10 | 1 | 3 | 5 | 10 | 1 | 3 | 5 | 10 | 2 | 3 | 5 | 10 |
|  | Toothbrush | 2 | 3 | 3 | 3 | 2 | 2 | 3 | 3 | 2 | 2 | 3 | 3 | 2 | 2 | 3 | 3 |
|  | Cotton swab | 0.07 | 0.29 | 0.43 | 1 | 0.07 | 0.14 | 0.43 | 1 | 0.07 | 0.14 | 0.43 | 1 | 0.07 | 0.14 | 0.43 | 1 |
|  | Towel | 2 | 3 | 3 | 5 | 2 | 3 | 4 | 5 | 2 | 3 | 3 | 5 | 2 | 3 | 3 | 5 |
|  | Handkerchief | 0.29 | 2 | 3 | 8 | 0.14 | 0.57 | 2 | 5 | 0.29 | 1 | 3 | 7 | 0.29 | 1 | 3 | 6 |
|  | Food tray | 0.71 | 1 | 1 | 3 | 0.71 | 0.71 | 1 | 3 | 0.71 | 1 | 1 | 3 | 0.71 | 1 | 1 | 3 |
|  | Lunch box | 0.01 | 0.02 | 0.03 | 0.14 | 0.01 | 0.03 | 0.03 | 0.29 | 0.01 | 0.02 | 0.03 | 0.14 | 0.01 | 0.02 | 0.03 | 0.14 |
|  | Water bottle | 0.29 | 1 | 3 | 5 | 0.29 | 0.71 | 2 | 5 | 0.29 | 1 | 3 | 5 | 0.29 | 0.86 | 2 | 5 |
|  | Car seat | 0.14 | 0.29 | 0.43 | 2 | 0.13 | 0.29 | 0.43 | 2 | 0.14 | 0.29 | 0.43 | 2 | 0.14 | 0.29 | 0.43 | 2 |
|  | Kid's chair | 1 | 2 | 3 | 4 | 1 | 2 | 2 | 4 | 1 | 2 | 2 | 4 | 1 | 2 | 3 | 4 |
| Sporting goods | Ball | 0.10 | 0.14 | 0.43 | 1 | 0.10 | 0.14 | 0.29 | 1 | 0.14 | 0.29 | 0.43 | 1 | 0.07 | 0.14 | 0.29 | 1 |
|  | Gloves | 0.07 | 0.14 | 0.29 | 1 | 0.07 | 0.10 | 0.14 | 0.43 | 0.07 | 0.14 | 0.29 | 0.57 | 0.07 | 0.14 | 0.29 | 1 |
|  | Bicycle | 0.14 | 0.29 | 0.43 | 1 | 0.10 | 0.14 | 0.29 | 1 | 0.14 | 0.29 | 0.43 | 1 | 0.13 | 0.29 | 0.43 | 1 |
|  | Inline skates | 0.07 | 0.14 | 0.29 | 0.57 | 0.03 | 0.14 | 0.14 | 0.43 | 0.07 | 0.14 | 0.29 | 0.43 | 0.07 | 0.14 | 0.29 | 0.43 |
|  | Roller shoes | 0.14 | 0.29 | 0.43 | 2 | 0.10 | 0.29 | 0.43 | 2 | 0.14 | 0.29 | 0.43 | 2 | 0.14 | 0.29 | 0.43 | 2 |
|  | Skateboard | 0.07 | 0.14 | 0.29 | 0.65 | 0.03 | 0.07 | 0.14 | 0.31 | 0.03 | 0.10 | 0.14 | 0.43 | 0.03 | 0.10 | 0.14 | 0.43 |
|  | Kick scooter | 0.14 | 0.29 | 0.43 | 1 | 0.10 | 0.14 | 0.29 | 1 | 0.14 | 0.29 | 0.43 | 1 | 0.13 | 0.29 | 0.43 | 1 |
|  | Picnic mat | 0.02 | 0.03 | 0.07 | 0.14 | 0.01 | 0.03 | 0.07 | 0.29 | 0.02 | 0.03 | 0.07 | 0.14 | 0.02 | 0.03 | 0.07 | 0.14 |
| Stationery | Oil pastel | 0.14 | 0.29 | 0.43 | 1 | 0.10 | 0.14 | 0.29 | 1 | 0.14 | 0.29 | 0.43 | 1 | 0.14 | 0.29 | 0.43 | 1 |
|  | Colored pencil | 0.14 | 0.29 | 0.57 | 2 | 0.14 | 0.29 | 0.43 | 1 | 0.14 | 0.29 | 0.43 | 2 | 0.14 | 0.29 | 0.43 | 2 |
|  | Paint supplies | 0.07 | 0.14 | 0.29 | 1 | 0.07 | 0.14 | 0.29 | 0.57 | 0.07 | 0.14 | 0.29 | 1 | 0.07 | 0.14 | 0.29 | 1 |
|  | Workbook | 0.29 | 0.43 | 1 | 2 | 0.29 | 0.43 | 0.86 | 2 | 0.29 | 0.43 | 1 | 2 | 0.29 | 0.43 | 1 | 2 |
|  | Sticker/sticker book | 0.14 | 0.29 | 0.43 | 1 | 0.07 | 0.14 | 0.29 | 1 | 0.10 | 0.14 | 0.29 | 1 | 0.13 | 0.29 | 0.43 | 1 |
|  | Notebook | NA | NA | NA | NA | NA | NA | NA | NA | NA | NA | NA | NA | NA | NA | NA | NA |
|  | Ballpoint pen | NA | NA | NA | NA | NA | NA | NA | NA | NA | NA | NA | NA | NA | NA | NA | NA |
|  | Pencil | NA | NA | NA | NA | NA | NA | NA | NA | NA | NA | NA | NA | NA | NA | NA | NA |
|  | Marker pen | NA | NA | NA | NA | NA | NA | NA | NA | NA | NA | NA | NA | NA | NA | NA | NA |
|  | Eraser | 2 | 4 | 6 | 20 | 3 | 4 | 6 | 12 | 3 | 4 | 6 | 15 | 3 | 4 | 6 | 15 |
|  | Correction tape/fluid | 1 | 2 | 3 | 10 | 2 | 2 | 3 | 5 | 2 | 2 | 3 | 6 | 2 | 2 | 3 | 6 |
|  | Glue | 2 | 3 | 4 | 10 | 2 | 3 | 5 | 10 | 2 | 3 | 4 | 10 | 2 | 3 | 4 | 10 |
|  | Adhesive | 1 | 1 | 3 | 10 | 1 | 2 | 3 | 10 | 1 | 2 | 3 | 7 | 1 | 2 | 3 | 10 |
|  | Scissors | 2 | 2 | 4 | 10 | 2 | 3 | 5 | 10 | 2 | 3 | 4 | 10 | 2 | 3 | 5 | 10 |

NA: Not available.

**Table S6.** Percentiles for use frequency (event/day) of children’s product by age group.

| Category | Product | 0-2 yrs | | | | 3-6 yrs | | | | 7-9 yrs | | | | 10-12 yrs | | | |
| --- | --- | --- | --- | --- | --- | --- | --- | --- | --- | --- | --- | --- | --- | --- | --- | --- | --- |
|  |  | 25p | 50p | 75p | 95p | 25p | 50p | 75p | 95p | 25p | 50p | 75p | 95p | 25p | 50p | 75p | 95p |
| Baby product | Self-righting toy | 0.29 | 1 | 2 | 4 | NA | NA | NA | NA | NA | NA | NA | NA | NA | NA | NA | NA |
|  | Baby rattle | 0.43 | 2 | 3 | 5 | NA | NA | NA | NA | NA | NA | NA | NA | NA | NA | NA | NA |
|  | Squeaky toy | 0.43 | 1 | 2 | 4 | NA | NA | NA | NA | NA | NA | NA | NA | NA | NA | NA | NA |
|  | Tactile toy | 0.43 | 1 | 2 | 4 | NA | NA | NA | NA | NA | NA | NA | NA | NA | NA | NA | NA |
|  | Baby mobile toy | 0.29 | 1 | 2 | 4 | NA | NA | NA | NA | NA | NA | NA | NA | NA | NA | NA | NA |
|  | Teether | 0.71 | 2 | 3 | 5 | NA | NA | NA | NA | NA | NA | NA | NA | NA | NA | NA | NA |
|  | Pacifier | 2 | 2 | 4 | 6 | NA | NA | NA | NA | NA | NA | NA | NA | NA | NA | NA | NA |
|  | Baby bouncer | 0.43 | 2 | 3 | 4 | NA | NA | NA | NA | NA | NA | NA | NA | NA | NA | NA | NA |
|  | Baby walker | 0.71 | 2 | 3 | 5 | NA | NA | NA | NA | NA | NA | NA | NA | NA | NA | NA | NA |
|  | Diaper | 5 | 6 | 8 | 10 | NA | NA | NA | NA | NA | NA | NA | NA | NA | NA | NA | NA |
|  | Teeth wipes | 1 | 1 | 2 | 3 | NA | NA | NA | NA | NA | NA | NA | NA | NA | NA | NA | NA |
|  | Baby bottle | 2 | 4 | 5 | 7 | NA | NA | NA | NA | NA | NA | NA | NA | NA | NA | NA | NA |
|  | Baby playpen | 0.43 | 1 | 3 | 4 | NA | NA | NA | NA | NA | NA | NA | NA | NA | NA | NA | NA |
| Toy | Play sand | 0.01 | 0.03 | 0.14 | 0.43 | 0.03 | 0.07 | 0.14 | 0.43 | 0.03 | 0.07 | 0.14 | 0.43 | 0.03 | 0.07 | 0.14 | 0.29 |
|  | Bubble-making toy | 0.02 | 0.03 | 0.14 | 0.29 | 0.02 | 0.03 | 0.10 | 0.29 | 0.02 | 0.03 | 0.13 | 0.43 | 0.01 | 0.03 | 0.07 | 0.29 |
|  | Kid's car | 0.29 | 0.57 | 2 | 3 | 0.14 | 0.43 | 1 | 3 | NA | NA | NA | NA | NA | NA | NA | NA |
|  | Kid's bike | 0.14 | 0.29 | 0.57 | 2 | 0.14 | 0.29 | 0.43 | 2 | NA | NA | NA | NA | NA | NA | NA | NA |
|  | Card game | NA | NA | NA | NA | 0.14 | 0.29 | 0.43 | 2 | 0.14 | 0.29 | 0.43 | 2 | 0.10 | 0.14 | 0.43 | 2 |
|  | Board game | NA | NA | NA | NA | 0.07 | 0.14 | 0.29 | 0.57 | 0.07 | 0.14 | 0.29 | 0.71 | 0.07 | 0.14 | 0.29 | 0.57 |
|  | Electronic game | NA | NA | NA | NA | 0.14 | 0.14 | 0.43 | 2 | 0.13 | 0.29 | 0.43 | 1 | 0.13 | 0.29 | 0.43 | 1 |
|  | Toy audio player | 0.43 | 1 | 2 | 3 | 0.29 | 0.43 | 1 | 2 | 0.07 | 0.29 | 0.43 | 1 | 0.07 | 0.14 | 0.32 | 1 |
|  | Toy video player | 0.29 | 1 | 2 | 3 | 0.14 | 0.29 | 1 | 2 | 0.07 | 0.14 | 0.29 | 1 | 0.10 | 0.14 | 0.29 | 1 |
|  | Beach ball | 0.02 | 0.07 | 0.29 | 1 | 0.02 | 0.03 | 0.07 | 0.29 | 0.02 | 0.03 | 0.07 | 0.29 | 0.02 | 0.03 | 0.07 | 0.29 |
|  | Swimming goggles | NA | NA | NA | NA | 0.02 | 0.03 | 0.10 | 0.43 | 0.02 | 0.07 | 0.29 | 0.43 | 0.02 | 0.03 | 0.14 | 0.43 |
|  | Bath toy | 0.29 | 0.57 | 1 | 1 | 0.14 | 0.29 | 0.43 | 1 | NA | NA | NA | NA | NA | NA | NA | NA |
| Daily product | Wet wipes | 4 | 5 | 10 | 15 | 2 | 3 | 5 | 8 | 0.57 | 2 | 3 | 5 | 0.57 | 2 | 3 | 5 |
|  | Toothbrush | 2 | 2 | 3 | 3 | 2 | 3 | 3 | 3 | 2 | 3 | 3 | 3 | 2 | 3 | 3 | 3 |
|  | Cotton swab | 0.14 | 0.29 | 0.71 | 1 | 0.07 | 0.14 | 0.43 | 1 | 0.07 | 0.14 | 0.29 | 1 | 0.07 | 0.14 | 0.29 | 1 |
|  | Towel | 2 | 3 | 4 | 6 | 2 | 3 | 3 | 5 | 2 | 3 | 4 | 5 | 2 | 3 | 3.25 | 5 |
|  | Handkerchief | 1 | 3 | 5 | 10 | 0.14 | 0.43 | 2 | 4 | 0.14 | 0.29 | 1 | 3 | 0.13 | 0.29 | 1 | 3 |
|  | Food tray | 1 | 1 | 2 | 3 | 0.71 | 1 | 2 | 3 | 0.71 | 0.71 | 1 | 2 | 0.71 | 0.71 | 1 | 2 |
|  | Lunch box | 0.01 | 0.02 | 0.03 | 0.68 | 0.01 | 0.02 | 0.03 | 0.16 | 0.01 | 0.02 | 0.03 | 0.14 | 0.01 | 0.03 | 0.03 | 0.14 |
|  | Water bottle | 0.71 | 2 | 4 | 6 | 0.29 | 1 | 3 | 5 | 0.29 | 0.71 | 1 | 4 | 0.29 | 0.71 | 1 | 4 |
|  | Car seat | 0.14 | 0.29 | 0.43 | 2 | 0.14 | 0.29 | 0.43 | 2 | NA | NA | NA | NA | NA | NA | NA | NA |
|  | Kid's chair | 1 | 2 | 3 | 4 | 0.61 | 2 | 2 | 3 | NA | NA | NA | NA | NA | NA | NA | NA |
| Sporting goods | Ball | 0.10 | 0.29 | 0.43 | 3 | 0.07 | 0.14 | 0.29 | 1 | 0.10 | 0.14 | 0.29 | 1 | 0.10 | 0.14 | 0.29 | 1 |
|  | Gloves | NA | NA | NA | NA | 0.03 | 0.14 | 0.14 | 0.43 | 0.07 | 0.14 | 0.29 | 0.57 | 0.07 | 0.14 | 0.29 | 0.57 |
|  | Bicycle | NA | NA | NA | NA | 0.14 | 0.29 | 0.43 | 1 | 0.14 | 0.29 | 0.43 | 1 | 0.14 | 0.29 | 0.43 | 1 |
|  | Inline skates | NA | NA | NA | NA | 0.07 | 0.14 | 0.29 | 0.43 | 0.07 | 0.14 | 0.29 | 0.57 | 0.07 | 0.14 | 0.29 | 0.43 |
|  | Roller shoes | NA | NA | NA | NA | 0.14 | 0.29 | 0.43 | 2 | 0.14 | 0.29 | 0.43 | 2 | 0.14 | 0.29 | 0.57 | 2 |
|  | Skateboard | NA | NA | NA | NA | 0.04 | 0.14 | 0.14 | 0.29 | 0.03 | 0.07 | 0.14 | 0.43 | 0.04 | 0.10 | 0.26 | 0.57 |
|  | Kick scooter | NA | NA | NA | NA | 0.14 | 0.29 | 0.43 | 1 | 0.13 | 0.29 | 0.43 | 1 | 0.07 | 0.14 | 0.29 | 1 |
|  | Picnic mat | 0.02 | 0.03 | 0.07 | 0.14 | 0.02 | 0.03 | 0.07 | 0.14 | 0.02 | 0.03 | 0.07 | 0.14 | 0.01 | 0.03 | 0.03 | 0.14 |
| Stationery | Oil pastel | 0.10 | 0.29 | 0.43 | 1 | 0.14 | 0.29 | 0.43 | 1 | 0.10 | 0.14 | 0.29 | 1 | 0.07 | 0.14 | 0.29 | 0.71 |
|  | Colored pencil | 0.14 | 0.29 | 0.57 | 2 | 0.16 | 0.29 | 0.57 | 2 | 0.14 | 0.29 | 0.43 | 1 | 0.14 | 0.14 | 0.29 | 1 |
|  | Paint supplies | 0.14 | 0.29 | 0.43 | 1 | 0.07 | 0.14 | 0.29 | 1 | 0.07 | 0.14 | 0.29 | 1 | 0.03 | 0.14 | 0.14 | 0.43 |
|  | Workbook | 0.14 | 0.29 | 0.50 | 2 | 0.14 | 0.43 | 1 | 2 | 0.29 | 0.43 | 1 | 2 | 0.29 | 0.71 | 1 | 3 |
|  | Sticker/sticker book | 0.14 | 0.29 | 0.43 | 1 | 0.14 | 0.29 | 0.43 | 1 | 0.07 | 0.14 | 0.29 | 1 | 0.07 | 0.14 | 0.29 | 1 |
|  | Notebook | NA | NA | NA | NA | NA | NA | NA | NA | NA | NA | NA | NA | NA | NA | NA | NA |
|  | Ballpoint pen | NA | NA | NA | NA | NA | NA | NA | NA | NA | NA | NA | NA | NA | NA | NA | NA |
|  | Pencil | NA | NA | NA | NA | NA | NA | NA | NA | NA | NA | NA | NA | NA | NA | NA | NA |
|  | Marker pen | NA | NA | NA | NA | NA | NA | NA | NA | NA | NA | NA | NA | NA | NA | NA | NA |
|  | Eraser | NA | NA | NA | NA | 2 | 4 | 5 | 10 | 3 | 4 | 6 | 20 | 3 | 4 | 7 | 20 |
|  | Correction tape/fluid | NA | NA | NA | NA | 1 | 2 | 3 | 5 | 1 | 2 | 3 | 6 | 2 | 2 | 3 | 6 |
|  | Glue | NA | NA | NA | NA | 2 | 3 | 5 | 10 | 2 | 3 | 4 | 10 | 2 | 2 | 4 | 10 |
|  | Adhesive | NA | NA | NA | NA | 1 | 2 | 3 | 10 | 1 | 2 | 3 | 6.40 | 1 | 2 | 3 | 6 |
|  | Scissors | NA | NA | NA | NA | 2 | 3 | 5 | 10 | 2 | 3 | 4 | 10 | 2 | 2 | 4 | 10 |

NA: Not available.

**Table S7.** Use durations (AM ± STD, min/event) of children’s product by gender for each season’s survey.

| Category | Product | Summer |  | Winter |  | Overall |  |  |
| --- | --- | --- | --- | --- | --- | --- | --- | --- |
|  |  | Boy | Girl | Boy | Girl | Boy | Girl | Significance |
| Baby product | Self-righting toy | 9.2±5.9 | 9.5±6.8 | 8.6±5.3 | 8.8±5.8 | 8.9±5.6 | 9.2±6.3 | ns |
|  | Baby rattle | 8.7±7.0 | 8.7±7.0 | 7.6±5.0 | 7.3±5.0 | 8.2±6.2 | 8.0±6.1 | ns |
|  | Squeaky toy | 11.1±7.9 | 11.5±8.7 | 11.4±6.7 | 11.3±7.6 | 11.3±7.3 | 11.4±8.2 | ns |
|  | Tactile toy | 10.7±8.1 | 10.9±8.0 | 12.1±7.7 | 12.3±8.4 | 11.4±7.9 | 11.6±8.2 | ns |
|  | Baby mobile toy | 8.5±5.8 | 8.5±6.1 | 10.7±8.3 | 9.9±6.6 | 9.4±7.0 | 9.1±6.3 | ns |
|  | Teether | 7.6±6.4 | 7.9±6.6 | 10.4±7.6 | 10.4±7.3 | 8.9±7.1 | 9.1±7.1 | ns |
|  | Pacifier | 17.7±12.3 | 17.8±11.6 | 16.9±10.2 | 17.9±12.0 | 17.3±11.4 | 17.8±11.8 | ns |
|  | Baby bouncer | 15.6±7.3 | 15.7±7.6 | 14.1±6.6 | 14.7±7.0 | 15.0±7.1 | 15.3±7.4 | ns |
|  | Baby walker | 18.9±8.6 | 19.3±9.1 | 15.1±7.6 | 16.1±8.9 | 17.4±8.5 | 18.0±9.1 | ns |
|  | Diaper | 183.5±118.6 | 185.2±131.7 | 187.7±91.6 | 188.8±90.4 | 185.5±106.3 | 186.9±113.4 | ns |
|  | Teeth wipes | 1.8±1.2 | 1.9±1.3 | 2.0±1.4 | 2.2±2.0 | 1.9±1.3 | 2.0±1.6 | ns |
|  | Baby bottle | 14.0±6.7 | 14.0±6.5 | 13.2±5.9 | 13.5±6.5 | 13.7±6.4 | 13.8±6.5 | ns |
|  | Baby playpen | 29.0±22.2 | 28.5±21.2 | 25.5±18.4 | 25.3±19.8 | 27.7±21.0 | 27.4±20.8 | ns |
| Toy | Play sand | 22.1±15.2 | 23.3±13.6 | 22.1±12.4 | 22.1±11.8 | 22.1±14.0 | 22.7±12.8 | ns |
|  | Bubble-making toy | 20.5±11.1 | 20.9±12.1 | 17.4±10.4 | 17.4±10.5 | 19.3±10.9 | 19.5±11.6 | ns |
|  | Kid's car | 18.3±10.4 | 17.9±10.0 | 16.3±9.2 | 15.4±8.9 | 17.4±9.9 | 16.8±9.6 | * |
|  | Kid's bike | 23.4±9.8 | 23.5±9.8 | 22.1±10.3 | 21.2±9.2 | 22.8±10.0 | 22.5±9.6 | ns |
|  | Card game | 23.1±12.4 | 23.5±12.7 | 22.8±11.9 | 22.7±12.0 | 23.0±12.2 | 23.2±12.4 | ns |
|  | Board game | 31.5±17.3 | 33.3±19.3 | 31.2±16.2 | 30.4±15.9 | 31.3±16.7 | 31.9±17.9 | ns |
|  | Electronic game | 35.6±22.1 | 33.9±20.4 | 35.2±21.3 | 29.7±17.5 | 35.4±21.7 | 31.7±19.1 | **** |
|  | Toy audio player | 15.3±8.6 | 15.8±9.2 | 14.9±8.5 | 14.7±8.4 | 15.1±8.6 | 15.3±8.9 | ns |
|  | Toy video player | 17.5±9.4 | 18.2±9.8 | 16.8±8.9 | 16.6±8.5 | 17.1±9.1 | 17.4±9.2 | ns |
|  | Beach ball | 28.3±20.8 | 28.4±21.8 | 19.6±14.5 | 19.0±13.6 | 26.2±19.8 | 26.2±20.5 | ns |
|  | Swimming goggles | 41.8±24.8 | 41.1±26.0 | 42.0±18.0 | 41.0±22.5 | 41.9±23.4 | 41.1±25.4 | ns |
|  | Bath toy | 16.2±9.3 | 16.3±8.7 | 13.8±8.1 | 13.5±8.3 | 15.2±8.9 | 15.1±8.6 | ns |
| Daily product | Wet wipes | NA | NA | NA | NA | NA | NA | - |
|  | Toothbrush | 2.8±1.2 | 2.8±1.2 | 2.7±1.3 | 2.7±1.3 | 2.8±1.3 | 2.7±1.3 | ns |
|  | Cotton swab | 1.8±1.6 | 1.8±1.5 | 2.0±1.8 | 1.9±1.5 | 1.9±1.7 | 1.9±1.5 | * |
|  | Towel | 3.4±2.9 | 3.5±3.3 | 3.4±3.5 | 3.4±3.4 | 3.4±3.2 | 3.4±3.4 | ns |
|  | Handkerchief | 4.9±16.0 | 5.3±16.2 | 7.5±21.5 | 7.7±26.0 | 6.2±18.9 | 6.5±21.7 | ns |
|  | Food tray | NA | NA | NA | NA | NA | NA | - |
|  | Lunch box | NA | NA | NA | NA | NA | NA | - |
|  | Water bottle | NA | NA | NA | NA | NA | NA | - |
|  | Car seat | 33.2±17.5 | 32.7±17.1 | 35.6±21.6 | 36.1±22.4 | 34.3±19.6 | 34.3±19.8 | ns |
|  | Kid's chair | 18.9±7.7 | 19.1±8.0 | 18.0±9.5 | 18.1±8.2 | 18.5±8.6 | 18.7±8.1 | ns |
| Sporting goods | Ball | 25.6±16.9 | 22.4±14.3 | 26.0±15.8 | 20.0±12.7 | 25.8±16.4 | 21.4±13.8 | **** |
|  | Gloves | 37.0±22.1 | 34.8±19.9 | 34.9±16.6 | 29.6±12.9 | 36.0±19.5 | 33.7±18.7 | * |
|  | Bicycle | 35.8±18.3 | 34.0±15.4 | 32.9±15.3 | 30.0±13.4 | 34.4±17.0 | 32.3±14.7 | **** |
|  | Inline skates | 36.6±19.4 | 35.3±15.8 | 33.4±15.9 | 31.8±13.5 | 35.0±17.8 | 33.6±14.8 | ** |
|  | Roller shoes | 43.6±32.8 | 44.7±40.5 | 42.9±38.8 | 44.2±50.2 | 43.4±35.1 | 44.5±44.9 | ns |
|  | Skateboard | 33.8±20.0 | 34.1±19.9 | 32.2±16.9 | 28.8±14.1 | 32.9±18.4 | 31.9±17.9 | ns |
|  | Kick scooter | 26.1±12.6 | 26.5±13.5 | 25.0±12.0 | 24.8±11.9 | 25.7±12.4 | 25.8±12.9 | ns |
|  | Picnic mat | 76.9±57.2 | 76.0±58.1 | 58.1±53.1 | 54.0±45.1 | 72.6±56.8 | 70.8±56.1 | ns |
| Stationery | Oil pastel | 22.6±13.9 | 22.8±14.2 | 21.6±10.6 | 21.1±10.3 | 22.1±12.5 | 22.0±12.5 | ns |
|  | Colored pencil | 19.5±10.1 | 20.2±10.5 | 17.0±9.2 | 17.6±9.6 | 18.3±9.8 | 18.9±10.2 | **** |
|  | Paint supplies | 23.0±14.9 | 24.1±14.4 | 20.1±11.1 | 21.7±11.7 | 22.0±13.8 | 23.3±13.5 | ** |
|  | Workbook | 24.5±11.8 | 25.1±12.5 | 23.8±11.6 | 23.8±11.7 | 24.2±11.7 | 24.4±12.1 | ns |
|  | Sticker/sticker book | 13.2±8.3 | 14.3±8.6 | 11.4±7.9 | 11.8±7.9 | 12.4±8.2 | 13.1±8.3 | **** |
|  | Notebook ^a^ | 26.5±25.2 | 27.4±26.4 | 25.9±20.7 | 26.3±21.7 | 26.2±23.1 | 26.8±24.2 | ns |
|  | Ballpoint pen ^a^ | 15.4±15.7 | 16.8±20.6 | 13.6±12.0 | 14.4±13.4 | 14.4±13.9 | 15.6±17.3 | ** |
|  | Pencil ^a^ | 29.2±28.3 | 30.2±30.3 | 28.1±25.4 | 28.2±24.4 | 28.6±26.9 | 29.2±27.6 | ns |
|  | Marker pen ^a^ | 15.6±17.1 | 17.1±16.5 | 11.1±7.2 | 11.7±8.1 | 13.6±13.8 | 14.8±13.8 | **** |
|  | Eraser | NA | NA | NA | NA | NA | NA | - |
|  | Correction tape/fluid | NA | NA | NA | NA | NA | NA | - |
|  | Glue | NA | NA | NA | NA | NA | NA | - |
|  | Adhesive | NA | NA | NA | NA | NA | NA | - |
|  | Scissors | NA | NA | NA | NA | NA | NA | - |

a: min/day, NA: Not available.

ns: p > 0.05, *: p ≤ 0.05, **: p ≤ 0.01, ***: p ≤ 0.001, ****: p ≤ 0.0001.

**Table S8.** Use durations (AM ± STD, min/event) of children’s product by age groups for each season’s survey.

| Category | Product | Summer |  |  |  | Winter |  |  |  | Overall |  |  |  |  |
| --- | --- | --- | --- | --- | --- | --- | --- | --- | --- | --- | --- | --- | --- | --- |
|  |  | 0-2 yrs | 3-6 yrs | 7-9 yrs | 10-12 yrs | 0-2 yrs | 3-6 yrs | 7-9 yrs | 10-12 yrs | 0-2 yrs | 3-6 yrs | 7-9 yrs | 10-12 yrs | Significance |
| Baby product | Self-righting toy | 9.3±6.3 | NA | NA | NA | 8.7±5.5 | NA | NA | NA | 9.0±6.0 | NA | NA | NA | - |
|  | Baby rattle | 8.7±7.0 | NA | NA | NA | 7.4±5.0 | NA | NA | NA | 8.1±6.2 | NA | NA | NA | - |
|  | Squeaky toy | 11.3±8.3 | NA | NA | NA | 11.4±7.2 | NA | NA | NA | 11.3±7.7 | NA | NA | NA | - |
|  | Tactile toy | 10.8±8.1 | NA | NA | NA | 12.2±8.1 | NA | NA | NA | 11.5±8.1 | NA | NA | NA | - |
|  | Baby mobile toy | 8.5±5.9 | NA | NA | NA | 10.4±7.5 | NA | NA | NA | 9.2±6.7 | NA | NA | NA | - |
|  | Teether | 7.7±6.5 | NA | NA | NA | 10.4±7.5 | NA | NA | NA | 9.0±7.1 | NA | NA | NA | - |
|  | Pacifier | 17.7±11.9 | NA | NA | NA | 17.4±11.2 | NA | NA | NA | 17.6±11.6 | NA | NA | NA | - |
|  | Baby bouncer | 15.6±7.4 | NA | NA | NA | 14.4±6.8 | NA | NA | NA | 15.2±7.2 | NA | NA | NA | - |
|  | Baby walker | 19.1±8.9 | NA | NA | NA | 15.6±8.3 | NA | NA | NA | 17.7±8.8 | NA | NA | NA | - |
|  | Diaper | 184.3±125.2 | NA | NA | NA | 188.2±91.0 | NA | NA | NA | 186.2±109.9 | NA | NA | NA | - |
|  | Teeth wipes | 1.9±1.2 | NA | NA | NA | 2.1±1.7 | NA | NA | NA | 2.0±1.5 | NA | NA | NA | - |
|  | Baby bottle | 14.0±6.6 | NA | NA | NA | 13.3±6.2 | NA | NA | NA | 13.7±6.5 | NA | NA | NA | - |
|  | Baby playpen | 28.8±21.7 | NA | NA | NA | 25.4±19.1 | NA | NA | NA | 27.6±20.9 | NA | NA | NA | - |
| Toy | Play sand | 14.6±8.2 | 22.5±12.9 | 29.9±19.3 | 26.5±10.8 | 20.7±12.9 | 22.3±12.0 | 23.5±11.5 | 22.1±11.4 | 17.4±11.1 | 22.4±12.5 | 27.0±16.5 | 24.4±11.3 | **** |
|  | Bubble-making toy | 16.3±9.0 | 21.1±10.0 | 23.8±15.0 | 22.7±12.8 | 14.7±9.7 | 17.7±10.1 | 18.3±9.5 | 20.6±12.8 | 15.6±9.4 | 19.8±10.2 | 21.7±13.5 | 21.9±12.8 | **** |
|  | Kid's car | 16.0±9.8 | 20.2±10.3 | NA | NA | 15.0±8.0 | 17.8±10.6 | NA | NA | 15.5±8.9 | 19.3±10.4 | NA | NA | **** |
|  | Kid's bike | 21.1±9.6 | 25.3±9.6 | NA | NA | 20.8±9.6 | 22.4±9.9 | NA | NA | 20.9±9.6 | 24.1±9.8 | NA | NA | **** |
|  | Card game | NA | 18.8±11.7 | 26.7±13.0 | 25.9±11.2 | NA | 19.6±10.8 | 24.8±12.4 | 25.8±11.9 | NA | 19.2±11.3 | 25.9±12.8 | 25.8±11.5 | **** |
|  | Board game | NA | 26.1±15.0 | 35.9±19.7 | 34.9±18.4 | NA | 26.0±13.8 | 32.9±17.1 | 34.4±16.0 | NA | 26.0±14.4 | 34.5±18.6 | 34.7±17.3 | **** |
|  | Electronic game | NA | 27.6±13.9 | 37.1±29.7 | 37.8±16.1 | NA | 24.2±10.9 | 34.8±17.1 | 40.3±25.4 | NA | 25.5±12.3 | 35.9±24.0 | 39.0±21.1 | **** |
|  | Toy audio player | 13.1±7.6 | 17.0±8.1 | 23.7±13.8 | 21.9±10.8 | 13.1±7.1 | 16.5±9.1 | 18.7±9.7 | 17.4±10.8 | 13.1±7.4 | 16.8±8.6 | 21.7±12.5 | 19.8±11.1 | **** |
|  | Toy video player | 14.3±7.4 | 19.0±8.5 | 25.0±13.9 | 25.7±12.9 | 14.7±7.6 | 18.0±8.5 | 21.6±11.6 | 21.2±11.7 | 14.5±7.5 | 18.6±8.5 | 23.7±13.1 | 23.6±12.6 | **** |
|  | Beach ball | 16.4±10.5 | 25.6±16.3 | 39.4±27.0 | 39.8±23.9 | 14.0±7.5 | 18.9±12.0 | 27.4±19.3 | 26.6±18.1 | 15.6±9.7 | 24.2±15.7 | 37.0±26.1 | 37.0±23.5 | **** |
|  | Swimming goggles | NA | 35.1±23.0 | 42.7±22.7 | 47.1±28.9 | NA | 35.1±16.1 | 45.5±19.2 | 44.7±23.5 | NA | 35.1±21.7 | 43.4±22.0 | 46.6±27.9 | **** |
|  | Bath toy | 13.5±6.6 | 19.8±10.4 | NA | NA | 12.2±7.4 | 15.9±8.8 | NA | NA | 12.9±7.0 | 18.3±9.9 | NA | NA | **** |
| Daily product | Wet wipes | NA | NA | NA | NA | NA | NA | NA | NA | NA | NA | NA | NA | - |
|  | Toothbrush | 2.3±1.1 | 2.8±1.0 | 3.0±1.2 | 3.2±1.5 | 2.6±1.6 | 2.6±1.0 | 2.9±1.4 | 2.9±1.3 | 2.4±1.4 | 2.7±1.0 | 2.9±1.3 | 3.0±1.5 | **** |
|  | Cotton swab | 1.7±1.6 | 1.9±1.8 | 1.8±1.1 | 1.9±1.6 | 1.9±1.7 | 2.0±1.9 | 2.0±1.5 | 1.9±1.5 | 1.8±1.6 | 1.9±1.8 | 1.9±1.4 | 1.9±1.5 | **** |
|  | Towel | 3.1±3.1 | 3.3±2.9 | 3.7±3.3 | 3.9±3.2 | 3.9±3.9 | 3.3±3.3 | 3.2±3.1 | 3.1±3.3 | 3.5±3.5 | 3.3±3.1 | 3.4±3.2 | 3.5±3.3 | **** |
|  | Handkerchief | 5.2±20.7 | 3.9±7.6 | 6.2±11.0 | 7.7±10.8 | 11.8±35.2 | 5.2±8.6 | 4.0±6.1 | 3.6±4.5 | 8.2±28.4 | 4.5±8.1 | 4.9±8.5 | 5.1±7.7 | **** |
|  | Food tray | NA | NA | NA | NA | NA | NA | NA | NA | NA | NA | NA | NA | - |
|  | Lunch box | NA | NA | NA | NA | NA | NA | NA | NA | NA | NA | NA | NA | - |
|  | Water bottle | NA | NA | NA | NA | NA | NA | NA | NA | NA | NA | NA | NA | - |
|  | Car seat | 31.9±15.5 | 34.1±19.2 | NA | NA | 35.6±21.9 | 36.2±22.1 | NA | NA | 33.7±19.0 | 35.0±20.5 | NA | NA | ** |
|  | Kid's chair | 18.3±8.0 | 20.7±7.1 | NA | NA | 17.6±9.4 | 19.3±7.4 | NA | NA | 18.0±8.7 | 20.2±7.3 | NA | NA | **** |
| Sporting goods | Ball | 12.6±8.1 | 21.9±11.7 | 32.4±17.1 | 35.1±17.3 | 13.5±8.1 | 20.9±11.1 | 30.3±15.0 | 34.5±17.0 | 13.0±8.1 | 21.5±11.5 | 31.4±16.2 | 34.8±17.2 | **** |
|  | Gloves | NA | 24.0±14.8 | 39.4±21.5 | 40.6±22.1 | NA | 27.5±13.4 | 35.1±15.7 | 38.1±17.3 | NA | 25.7±14.3 | 37.5±19.2 | 39.5±20.1 | **** |
|  | Bicycle | NA | 30.7±15.6 | 35.0±16.0 | 38.8±18.4 | NA | 28.7±12.9 | 32.6±15.0 | 33.5±15.2 | NA | 29.8±14.5 | 33.9±15.6 | 36.3±17.2 | **** |
|  | Inline skates | NA | 32.5±18.8 | 35.4±16.5 | 38.2±18.0 | NA | 28.0±12.9 | 34.4±15.3 | 35.0±14.7 | NA | 29.7±15.6 | 34.9±15.9 | 36.7±16.7 | **** |
|  | Roller shoes | NA | 40.3±35.8 | 45.0±35.2 | 44.4±38.4 | NA | 31.6±22.8 | 43.3±41.7 | 50.6±56.7 | NA | 35.6±29.7 | 44.3±38.1 | 46.5±45.5 | **** |
|  | Skateboard | NA | 36.7±27.1 | 29.9±15.6 | 35.7±20.5 | NA | 25.6±14.1 | 32.5±18.7 | 33.2±15.0 | NA | 28.4±18.9 | 31.2±17.2 | 34.5±18.2 | **** |
|  | Kick scooter | NA | 23.5±11.5 | 30.5±14.7 | 30.2±13.1 | NA | 23.8±11.6 | 26.4±12.7 | 26.6±11.5 | NA | 23.7±11.5 | 28.8±14.0 | 28.7±12.6 | **** |
|  | Picnic mat | 71.3±50.5 | 74.7±54.4 | 79.2±62.6 | 85.4±67.3 | 50.2±38.9 | 55.3±47.3 | 56.5±56.4 | 63.5±54.6 | 66.7±49.0 | 70.3±53.5 | 73.8±62.0 | 79.5±64.9 | **** |
| Stationery | Oil pastel | 13.9±7.7 | 21.9±16.0 | 26.6±11.9 | 26.3±12.6 | 14.3±8.6 | 20.6±9.9 | 23.9±10.6 | 24.1±10.4 | 14.1±8.1 | 21.2±13.4 | 25.3±11.4 | 25.3±11.7 | **** |
|  | Colored pencil | 13.2±7.4 | 19.1±9.1 | 23.1±10.7 | 22.9±11.0 | 12.4±7.1 | 17.2±8.7 | 19.4±9.7 | 19.5±10.5 | 12.8±7.3 | 18.2±8.9 | 21.3±10.4 | 21.4±10.9 | **** |
|  | Paint supplies | 13.6±6.6 | 22.8±16.3 | 27.5±13.0 | 26.8±14.2 | 17.9±10.6 | 20.1±10.7 | 21.9±11.8 | 22.3±12.3 | 14.5±7.8 | 21.8±14.5 | 25.6±12.9 | 25.1±13.7 | **** |
|  | Workbook | 15.4±7.7 | 20.0±8.8 | 27.1±11.8 | 31.6±13.2 | 16.0±7.9 | 20.1±8.5 | 26.0±11.7 | 29.3±13.2 | 15.7±7.8 | 20.0±8.6 | 26.5±11.8 | 30.5±13.2 | **** |
|  | Sticker/sticker book | 11.0±6.4 | 13.6±8.3 | 15.8±9.1 | 15.6±9.5 | 9.2±5.7 | 11.9±7.7 | 12.8±8.7 | 12.5±9.1 | 10.2±6.1 | 12.8±8.1 | 14.3±9.1 | 14.1±9.4 | **** |
|  | Notebook ^a^ | NA | 17.9±18.4 | 30.1±20.7 | 36.8±33.9 | NA | 19.1±17.1 | 28.2±19.1 | 33.3±25.0 | NA | 18.5±17.8 | 29.1±20.0 | 35.1±29.8 | **** |
|  | Ballpoint pen ^a^ | NA | 9.8±8.4 | 16.5±18.0 | 20.5±22.2 | NA | 9.7±6.2 | 13.8±11.6 | 16.3±15.2 | NA | 9.8±7.5 | 15.0±14.9 | 18.2±18.9 | **** |
|  | Pencil ^a^ | NA | 18.0±10.0 | 34.8±30.1 | 42.3±39.7 | NA | 18.5±11.0 | 31.3±22.1 | 38.7±34.9 | NA | 18.3±10.5 | 33.0±26.4 | 40.5±37.4 | **** |
|  | Marker pen ^a^ | NA | 12.6±13.1 | 18.0±16.6 | 19.6±19.8 | NA | 10.7±6.5 | 11.7±7.7 | 11.9±8.8 | NA | 11.8±10.8 | 15.2±13.7 | 16.2±16.4 | **** |
|  | Eraser | NA | NA | NA | NA | NA | NA | NA | NA | NA | NA | NA | NA | - |
|  | Correction tape/fluid | NA | NA | NA | NA | NA | NA | NA | NA | NA | NA | NA | NA | - |
|  | Glue | NA | NA | NA | NA | NA | NA | NA | NA | NA | NA | NA | NA | - |
|  | Adhesive | NA | NA | NA | NA | NA | NA | NA | NA | NA | NA | NA | NA | - |
|  | Scissors | NA | NA | NA | NA | NA | NA | NA | NA | NA | NA | NA | NA | - |

a: min/day, NA: Not available.

ns: p > 0.05, *: p ≤ 0.05, **: p ≤ 0.01, ***: p ≤ 0.001, ****: p ≤ 0.0001

**Table S9.** Percentiles for use duration (min/event) of children’s product by season and gender.

| Category | Product | Season | | | | | | | | Gender | | | | | | | |
| --- | --- | --- | --- | --- | --- | --- | --- | --- | --- | --- | --- | --- | --- | --- | --- | --- | --- |
|  |  | Summer | | | | Winter | | | | Boy | | | | Girl | | | |
|  |  | 25p | 50p | 75p | 95p | 25p | 50p | 75p | 95p | 25p | 50p | 75p | 95p | 25p | 50p | 75p | 95p |
| Baby product | Self-righting toy | 5 | 10 | 10 | 20 | 5 | 10 | 10 | 20 | 5 | 10 | 10 | 20 | 5 | 10 | 10 | 20 |
|  | Baby rattle | 5 | 5 | 10 | 20 | 5 | 5 | 10 | 20 | 5 | 5 | 10 | 20 | 5 | 5 | 10 | 20 |
|  | Squeaky toy | 5 | 10 | 10 | 30 | 5 | 10 | 15 | 21.5 | 5 | 10 | 15 | 30 | 5 | 10 | 15 | 30 |
|  | Tactile toy | 5 | 5 | 10 | 20 | 5 | 10 | 10 | 20 | 5 | 10 | 10 | 20 | 5 | 10 | 10 | 20 |
|  | Baby mobile toy | 5 | 10 | 10 | 30 | 7 | 10 | 15 | 30 | 5 | 10 | 15 | 30 | 5 | 10 | 15 | 30 |
|  | Teether | 5 | 5 | 10 | 20 | 5 | 10 | 15 | 20 | 5 | 5 | 10 | 20 | 5 | 5 | 10 | 20 |
|  | Pacifier | 10 | 15 | 20 | 30 | 10 | 15 | 20 | 30 | 10 | 15 | 20 | 30 | 10 | 15 | 20 | 30 |
|  | Baby bouncer | 10 | 15 | 20 | 30 | 10 | 10 | 20 | 30 | 10 | 15 | 20 | 30 | 10 | 15 | 20 | 30 |
|  | Baby walker | 10 | 20 | 20 | 30 | 10 | 15 | 20 | 30 | 10 | 20 | 20 | 30 | 10 | 20 | 20 | 30 |
|  | Diaper | 120 | 150 | 180 | 600 | 120 | 180 | 240 | 360 | 120 | 180 | 200 | 420 | 120 | 160 | 210 | 480 |
|  | Teeth wipes | 1 | 1 | 2 | 5 | 1 | 2 | 2 | 5 | 1 | 1 | 2 | 5 | 1 | 1 | 2 | 5 |
|  | Baby bottle | 10 | 10 | 20 | 30 | 10 | 10 | 15 | 30 | 10 | 10 | 20 | 30 | 10 | 10 | 19 | 30 |
|  | Baby playpen | 15 | 30 | 30 | 60 | 15 | 20 | 30 | 60 | 15 | 20 | 30 | 60 | 15 | 20 | 30 | 60 |
| Toy | Play sand | 10 | 20 | 30 | 40 | 15 | 20 | 30 | 50 | 10 | 20 | 30 | 40 | 15 | 20 | 30 | 40 |
|  | Bubble-making toy | 10 | 20 | 30 | 40 | 10 | 15 | 20 | 30 | 10 | 20 | 30 | 30 | 10 | 20 | 30 | 40 |
|  | Kid's car | 10 | 17 | 25 | 33 | 10 | 15 | 20 | 30 | 10 | 15 | 20 | 30 | 10 | 15 | 20 | 30 |
|  | Kid's bike | 20 | 20 | 30 | 40 | 15 | 20 | 30 | 40 | 15 | 20 | 30 | 40 | 15 | 20 | 30 | 40 |
|  | Card game | 15 | 20 | 30 | 50 | 15 | 20 | 30 | 47 | 15 | 20 | 30 | 40 | 15 | 20 | 30 | 50 |
|  | Board game | 20 | 30 | 40 | 60 | 20 | 30 | 40 | 60 | 20 | 30 | 40 | 60 | 20 | 30 | 40 | 60 |
|  | Electronic game | 20 | 30 | 40 | 60 | 20 | 30 | 40 | 60 | 20 | 30 | 40 | 60 | 20 | 30 | 40 | 60 |
|  | Toy audio player | 10 | 15 | 20 | 30 | 10 | 10 | 20 | 30 | 10 | 15 | 20 | 30 | 10 | 10 | 20 | 30 |
|  | Toy video player | 10 | 20 | 20 | 30 | 10 | 15 | 20 | 30 | 10 | 15 | 20 | 30 | 10 | 15 | 20 | 30 |
|  | Beach ball | 15 | 25 | 30 | 60 | 10 | 15 | 25 | 50 | 10 | 20 | 30 | 60 | 10 | 20 | 30 | 60 |
|  | Swimming goggles | 30 | 30 | 50 | 60 | 30 | 40 | 50 | 60 | 30 | 40 | 60 | 60 | 30 | 35 | 50 | 60 |
|  | Bath toy | 10 | 15 | 20 | 30 | 10 | 10 | 20 | 30 | 10 | 10 | 20 | 30 | 10 | 10 | 20 | 30 |
| Daily product | Wet wipes | NA | NA | NA | NA | NA | NA | NA | NA | NA | NA | NA | NA | NA | NA | NA | NA |
|  | Toothbrush | 2 | 3 | 3 | 5 | 2 | 3 | 3 | 5 | 2 | 3 | 3 | 5 | 2 | 3 | 3 | 5 |
|  | Cotton swab | 1 | 1 | 2 | 5 | 1 | 1 | 2 | 5 | 1 | 1 | 2 | 5 | 1 | 1 | 2 | 5 |
|  | Towel | 2 | 2 | 5 | 10 | 2 | 2 | 4 | 10 | 2 | 2 | 5 | 10 | 2 | 2 | 5 | 10 |
|  | Handkerchief | 1 | 1 | 3 | 20 | 1 | 2 | 5 | 30 | 1 | 2 | 5 | 20 | 1 | 2 | 5 | 24.7 |
|  | Food tray | NA | NA | NA | NA | NA | NA | NA | NA | NA | NA | NA | NA | NA | NA | NA | NA |
|  | Lunch box | NA | NA | NA | NA | NA | NA | NA | NA | NA | NA | NA | NA | NA | NA | NA | NA |
|  | Water bottle | NA | NA | NA | NA | NA | NA | NA | NA | NA | NA | NA | NA | NA | NA | NA | NA |
|  | Car seat | 20 | 30 | 40 | 60 | 20 | 30 | 40 | 60 | 20 | 30 | 40 | 60 | 20 | 30 | 40 | 60 |
|  | Kid's chair | 15 | 20 | 20 | 30 | 10 | 20 | 20 | 30 | 10 | 20 | 20 | 30 | 10 | 20 | 20 | 30 |
| Sporting goods | Ball | 10 | 20 | 30 | 60 | 10 | 20 | 30 | 60 | 15 | 20 | 30 | 60 | 10 | 20 | 30 | 50 |
|  | Gloves | 20 | 30 | 50 | 60 | 20 | 30 | 40 | 60 | 20 | 30 | 45 | 60 | 20 | 30 | 40 | 60 |
|  | Bicycle | 30 | 30 | 40 | 60 | 20 | 30 | 40 | 60 | 25 | 30 | 40 | 60 | 20 | 30 | 40 | 60 |
|  | Inline skates | 30 | 30 | 40 | 60 | 20 | 30 | 40 | 60 | 25 | 30 | 40 | 60 | 20 | 30 | 40 | 60 |
|  | Roller shoes | 30 | 30 | 60 | 90 | 20 | 30 | 50 | 120 | 30 | 30 | 50 | 120 | 20 | 30 | 60 | 120 |
|  | Skateboard | 20 | 30 | 40 | 60 | 20 | 30 | 40 | 60 | 20 | 30 | 40 | 60 | 20 | 30 | 35 | 60 |
|  | Kick scooter | 20 | 20 | 30 | 60 | 20 | 20 | 30 | 50 | 20 | 20 | 30 | 50 | 20 | 20 | 30 | 50 |
|  | Picnic mat | 35 | 60 | 120 | 180 | 30 | 40 | 60 | 130 | 30 | 60 | 100 | 180 | 30 | 60 | 100 | 180 |
| Stationery | Oil pastel | 15 | 20 | 30 | 40 | 10 | 20 | 30 | 40 | 15 | 20 | 30 | 40 | 10 | 20 | 30 | 40 |
|  | Colored pencil | 10 | 20 | 30 | 35 | 10 | 15 | 20 | 30 | 10 | 20 | 20 | 30 | 10 | 20 | 25 | 30 |
|  | Paint supplies | 15 | 20 | 30 | 50 | 10 | 20 | 30 | 40 | 10 | 20 | 30 | 40 | 15 | 20 | 30 | 50 |
|  | Workbook | 15 | 20 | 30 | 50 | 15 | 20 | 30 | 50 | 15 | 20 | 30 | 50 | 15 | 20 | 30 | 50 |
|  | Sticker/sticker book | 10 | 10 | 20 | 30 | 5 | 10 | 15 | 30 | 5 | 10 | 15 | 30 | 10 | 10 | 20 | 30 |
|  | Notebook ^a^ | 15 | 20 | 30 | 60 | 15 | 20 | 30 | 60 | 15 | 20 | 30 | 60 | 15 | 20 | 30 | 60 |
|  | Ballpoint pen ^a^ | 10 | 10 | 20 | 40 | 5 | 10 | 20 | 30 | 5 | 10 | 20 | 30 | 10 | 10 | 20 | 40 |
|  | Pencil ^a^ | 15 | 20 | 30 | 60 | 15 | 20 | 30 | 60 | 15 | 20 | 30 | 60 | 15 | 20 | 30 | 60 |
|  | Marker pen ^a^ | 10 | 10 | 20 | 35 | 5 | 10 | 15 | 30 | 8 | 10 | 20 | 30 | 10 | 10 | 20 | 30 |
|  | Eraser | NA | NA | NA | NA | NA | NA | NA | NA | NA | NA | NA | NA | NA | NA | NA | NA |
|  | Correction tape/fluid | NA | NA | NA | NA | NA | NA | NA | NA | NA | NA | NA | NA | NA | NA | NA | NA |
|  | Glue | NA | NA | NA | NA | NA | NA | NA | NA | NA | NA | NA | NA | NA | NA | NA | NA |
|  | Adhesive | NA | NA | NA | NA | NA | NA | NA | NA | NA | NA | NA | NA | NA | NA | NA | NA |
|  | Scissors | NA | NA | NA | NA | NA | NA | NA | NA | NA | NA | NA | NA | NA | NA | NA | NA |

a: min/day, NA: Not available.

**Table S10.** Percentiles for use duration (min/event) of children’s product by age group.

| Category | Product | 0-2 yrs | | | | 3-6 yrs | | | | 7-9 yrs | | | | 10-12 yrs | | | |
| --- | --- | --- | --- | --- | --- | --- | --- | --- | --- | --- | --- | --- | --- | --- | --- | --- | --- |
|  |  | 25p | 50p | 75p | 95p | 25p | 50p | 75p | 95p | 25p | 50p | 75p | 95p | 25p | 50p | 75p | 95p |
| Baby product | Self-righting toy | 5 | 10 | 10 | 20 | NA | NA | NA | NA | NA | NA | NA | NA | NA | NA | NA | NA |
|  | Baby rattle | 5 | 5 | 10 | 20 | NA | NA | NA | NA | NA | NA | NA | NA | NA | NA | NA | NA |
|  | Squeaky toy | 5 | 10 | 15 | 30 | NA | NA | NA | NA | NA | NA | NA | NA | NA | NA | NA | NA |
|  | Tactile toy | 5 | 10 | 10 | 20 | NA | NA | NA | NA | NA | NA | NA | NA | NA | NA | NA | NA |
|  | Baby mobile toy | 5 | 10 | 15 | 30 | NA | NA | NA | NA | NA | NA | NA | NA | NA | NA | NA | NA |
|  | Teether | 5 | 5 | 10 | 20 | NA | NA | NA | NA | NA | NA | NA | NA | NA | NA | NA | NA |
|  | Pacifier | 10 | 15 | 20 | 30 | NA | NA | NA | NA | NA | NA | NA | NA | NA | NA | NA | NA |
|  | Baby bouncer | 10 | 15 | 20 | 30 | NA | NA | NA | NA | NA | NA | NA | NA | NA | NA | NA | NA |
|  | Baby walker | 10 | 20 | 20 | 30 | NA | NA | NA | NA | NA | NA | NA | NA | NA | NA | NA | NA |
|  | Diaper | 120 | 170 | 200 | 480 | NA | NA | NA | NA | NA | NA | NA | NA | NA | NA | NA | NA |
|  | Teeth wipes | 1 | 1 | 2 | 5 | NA | NA | NA | NA | NA | NA | NA | NA | NA | NA | NA | NA |
|  | Baby bottle | 10 | 10 | 20 | 30 | NA | NA | NA | NA | NA | NA | NA | NA | NA | NA | NA | NA |
|  | Baby playpen | 15 | 20 | 30 | 60 | NA | NA | NA | NA | NA | NA | NA | NA | NA | NA | NA | NA |
| Toy | Play sand | 10 | 15 | 20 | 30 | 15 | 20 | 30 | 40 | 20 | 20 | 30 | 60 | 20 | 20 | 30 | 40 |
|  | Bubble-making toy | 10 | 15 | 20 | 30 | 10 | 20 | 30 | 40 | 10 | 20 | 30 | 40 | 10 | 20 | 30 | 60 |
|  | Kid's car | 10 | 10 | 20 | 30 | 10 | 20 | 30 | 35 | NA | NA | NA | NA | NA | NA | NA | NA |
|  | Kid's bike | 15 | 20 | 30 | 35 | 20 | 20 | 30 | 40 | NA | NA | NA | NA | NA | NA | NA | NA |
|  | Card game | NA | NA | NA | NA | 10 | 20 | 20 | 30 | 20 | 20 | 30 | 50 | 20 | 25 | 30 | 50 |
|  | Board game | NA | NA | NA | NA | 15 | 20 | 30 | 60 | 20 | 30 | 40 | 60 | 20 | 30 | 40 | 60 |
|  | Electronic game | NA | NA | NA | NA | 20 | 20 | 30 | 50 | 20 | 30 | 40 | 60 | 30 | 30 | 50 | 60 |
|  | Toy audio player | 10 | 10 | 20 | 30 | 10 | 15 | 20 | 30 | 10 | 20 | 30 | 40 | 10 | 20 | 30 | 40 |
|  | Toy video player | 10 | 10 | 20 | 30 | 10 | 20 | 20 | 30 | 15 | 20 | 30 | 53.5 | 20 | 20 | 30 | 50 |
|  | Beach ball | 10 | 10 | 20 | 30 | 15 | 20 | 30 | 50 | 20 | 30 | 40 | 91.5 | 20 | 30 | 50 | 60 |
|  | Swimming goggles | NA | NA | NA | NA | 20 | 30 | 40 | 60 | 30 | 40 | 60 | 60 | 30 | 40 | 60 | 90 |
|  | Bath toy | 10 | 10 | 15 | 30 | 10 | 20 | 20 | 30 | NA | NA | NA | NA | NA | NA | NA | NA |
| Daily product | Wet wipes | NA | NA | NA | NA | NA | NA | NA | NA | NA | NA | NA | NA | NA | NA | NA | NA |
|  | Toothbrush | 1.5 | 2 | 3 | 5 | 2 | 3 | 3 | 5 | 2 | 3 | 3 | 5 | 2 | 3 | 3 | 5 |
|  | Cotton swab | 1 | 1 | 2 | 5 | 1 | 1 | 2 | 5 | 1 | 1 | 2 | 5 | 1 | 1 | 2 | 5 |
|  | Towel | 1 | 2 | 5 | 10 | 2 | 2 | 4 | 10 | 2 | 3 | 5 | 10 | 2 | 3 | 5 | 10 |
|  | Handkerchief | 1 | 2 | 5 | 30 | 1 | 2 | 5 | 20 | 1 | 2 | 5 | 20 | 1 | 3 | 5 | 20 |
|  | Food tray | NA | NA | NA | NA | NA | NA | NA | NA | NA | NA | NA | NA | NA | NA | NA | NA |
|  | Lunch box | NA | NA | NA | NA | NA | NA | NA | NA | NA | NA | NA | NA | NA | NA | NA | NA |
|  | Water bottle | NA | NA | NA | NA | NA | NA | NA | NA | NA | NA | NA | NA | NA | NA | NA | NA |
|  | Car seat | 20 | 30 | 40 | 60 | 20 | 30 | 40 | 60 | NA | NA | NA | NA | NA | NA | NA | NA |
|  | Kid's chair | 10 | 20 | 20 | 30 | 15 | 20 | 23 | 30 | NA | NA | NA | NA | NA | NA | NA | NA |
| Sporting goods | Ball | 10 | 10 | 15 | 30 | 10 | 20 | 30 | 40 | 20 | 30 | 40 | 60 | 30 | 30 | 40 | 60 |
|  | Gloves | NA | NA | NA | NA | 20 | 20 | 30 | 60 | 30 | 30 | 50 | 60 | 30 | 30 | 50 | 60 |
|  | Bicycle | NA | NA | NA | NA | 20 | 30 | 30 | 60 | 20 | 30 | 40 | 60 | 30 | 30 | 40 | 60 |
|  | Inline skates | NA | NA | NA | NA | 20 | 30 | 30 | 60 | 30 | 30 | 40 | 60 | 30 | 30 | 40 | 60 |
|  | Roller shoes | NA | NA | NA | NA | 20 | 30 | 35 | 120 | 30 | 30 | 50 | 120 | 30 | 30 | 60 | 120 |
|  | Skateboard | NA | NA | NA | NA | 20 | 27.5 | 30 | 60 | 20 | 30 | 40 | 60 | 20 | 30 | 40 | 60 |
|  | Kick scooter | NA | NA | NA | NA | 15 | 20 | 30 | 40 | 20 | 30 | 30 | 60 | 20 | 30 | 30 | 60 |
|  | Picnic mat | 30 | 60 | 100 | 180 | 30 | 60 | 100 | 180 | 30 | 60 | 100 | 180 | 40 | 60 | 120 | 200 |
| Stationery | Oil pastel | 10 | 10 | 20 | 30 | 10 | 20 | 30 | 40 | 20 | 20 | 30 | 50 | 20 | 25 | 30 | 50 |
|  | Colored pencil | 10 | 10 | 15 | 30 | 10 | 20 | 20 | 30 | 10 | 20 | 30 | 40 | 10 | 20 | 30 | 40 |
|  | Paint supplies | 10 | 10 | 20 | 30 | 15 | 20 | 30 | 31.75 | 20 | 20 | 30 | 60 | 15 | 20 | 30 | 50 |
|  | Workbook | 10 | 15 | 20 | 30 | 15 | 20 | 25 | 30 | 20 | 30 | 30 | 50 | 20 | 30 | 40 | 60 |
|  | Sticker/sticker book | 5 | 10 | 10 | 20 | 10 | 10 | 15 | 30 | 10 | 10 | 20 | 30 | 10 | 10 | 20 | 30 |
|  | Notebook ^a^ | NA | NA | NA | NA | 10 | 15 | 20 | 30 | 20 | 30 | 30 | 60 | 20 | 30 | 40 | 60 |
|  | Ballpoint pen ^a^ | NA | NA | NA | NA | 5 | 10 | 10 | 20 | 10 | 10 | 20 | 30 | 10 | 10 | 20 | 50 |
|  | Pencil ^a^ | NA | NA | NA | NA | 10 | 20 | 20 | 30 | 20 | 30 | 30 | 60 | 20 | 30 | 50 | 100 |
|  | Marker pen ^a^ | NA | NA | NA | NA | 5 | 10 | 15 | 27 | 10 | 10 | 20 | 30 | 10 | 10 | 20 | 40 |
|  | Eraser | NA | NA | NA | NA | NA | NA | NA | NA | NA | NA | NA | NA | NA | NA | NA | NA |
|  | Correction tape/fluid | NA | NA | NA | NA | NA | NA | NA | NA | NA | NA | NA | NA | NA | NA | NA | NA |
|  | Glue | NA | NA | NA | NA | NA | NA | NA | NA | NA | NA | NA | NA | NA | NA | NA | NA |
|  | Adhesive | NA | NA | NA | NA | NA | NA | NA | NA | NA | NA | NA | NA | NA | NA | NA | NA |
|  | Scissors | NA | NA | NA | NA | NA | NA | NA | NA | NA | NA | NA | NA | NA | NA | NA | NA |

a: min/day, NA: Not available.
